# Taxonomic classification of strain PO100/5 shows a broader geographic distribution and genetic markers of the recently described *Corynebacterium silvaticum*

**DOI:** 10.1371/journal.pone.0244210

**Published:** 2020-12-21

**Authors:** Marcus Vinicius Canário Viana, Rodrigo Profeta, Alessandra Lima da Silva, Raquel Hurtado, Janaína Canário Cerqueira, Bruna Ferreira Sampaio Ribeiro, Marcelle Oliveira Almeida, Francielly Morais-Rodrigues, Siomar de Castro Soares, Manuela Oliveira, Luís Tavares, Henrique Figueiredo, Alice Rebecca Wattam, Debmalya Barh, Preetam Ghosh, Artur Silva, Vasco Azevedo

**Affiliations:** 1 Department of Genetics, Ecology and Evolution, Institute of Biological Sciences, Federal University of Minas Gerais, Belo Horizonte, Minas Gerais, Brazil; 2 Department of Genetics, Institute of Biological Sciences, Federal University of Pará, Belém, Pará, Brazil; 3 Department of Immunology, Microbiology and Parasitology, Institute of Biological Sciences and Natural Sciences, Federal University of Triângulo Mineiro, Uberaba, Minas Gerais, Brazil; 4 Centre for Interdisciplinary Research in Animal Health, Faculty of Veterinary Medicine, University of Lisbon, Lisboa, Portugal; 5 National Reference Laboratory of Aquatic Animal Disease, Federal University of Minas Gerais, Belo Horizonte, Minas Gerais, Brazil; 6 Biocomplexity Institute, University of Virginia, Charlottesville, Virginia, United States of America; 7 Institute of Integrative Omics and Applied Biotechnology, Purba Medinipur, West Bengal, India; 8 Department of Computer Science, Virginia Commonwealth University, Richmond, Virginia, United States of America; Academia Sinica, TAIWAN

## Abstract

The bacterial strain PO100/5 was isolated from a skin abscess taken from a pig (*Sus scrofa domesticus*) in the Alentejo region of southern Portugal. It was identified as *Corynebacterium pseudotuberculosis* using biochemical tests, multiplex PCR and Pulsed Field Gel Electrophoresis. After genome sequencing and *rpoB* phylogeny, the strain was classified as *C*. *ulcerans*. To better understand the taxonomy of this strain and improve identification methods, we compared strain PO100/5 to other publicly available genomes from *C*. *diphtheriae* group. Taxonomic analysis reclassified it and three others strains as the recently described *C*. *silvaticum*, which have been isolated from wild boar and roe deer in Germany and Austria. The results showed that PO100/5 is the first sequenced genome of a *C*. *silvaticum* strain from livestock and a different geographical region, has the unique sequence type ST709, and could be could produce the *diphtheriae* toxin, along with strain 05–13. Genomic analysis of PO100/5 showed four prophages, and eight conserved genomic islands in comparison to *C*. *ulcerans*. Pangenome analysis of 38 *C*. *silvaticum* and 76 *C*. *ulcerans* genomes suggested that C. *silvaticum* is a genetically homogeneous species, with 73.6% of its genes conserved and a pangenome near to be closed (α > 0.952). There are 172 genes that are unique to *C*. *silvaticum* in comparison to *C*. *ulcerans*. Most of these conserved genes are related to nutrient uptake and metabolism, prophages or immunity against them, and could be genetic markers for species identification. Strains PO100/5 (livestock) and KL0182^T^ (wild boar) were predicted to be potential human pathogens. This information may be useful for identification and surveillance of this pathogen.

## Introduction

The genus *Corynebacterium* from the phylum Actinobacteria has Gram-positive bacteria of biotechnological, veterinary and medical relevance with free, commensal and pathogenic lifestyles. Within pathogenic species, the most prominent species are the nearly exclusively human pathogen *C*. *diphtheriae* and the zoonotic *C*. *pseudotuberculosis* and *C*. *ulcerans*. These three compose the *C*. *diphtheriae* group, a clade of species that can be lysogenized by phages harboring the diphtheria toxin (DT) gene (*tox*) [1]. Within this group, three new species were recently described. *C*. *rouxii* [2] and *C*. *belfantii* are reclassifications of some of the *C*. *diphtheriae* biovar Belfanti strains [3]. *C*. *belfantii* is also a synonym of *C*. *diphtheriae* subspecies *lausannense* [2]. *C*. *silvaticum* [4] is a reclassification of atypical *C*. *ulcerans* strains. Strains of *C*. *silvaticum* were previously described as atypical non-toxigenic but *tox*-gene-bearing (NTTB) strains of *C*. *ulcerans*, isolated from wild boars and roe deer in Germany and Austria, which caused caseous lymphadenitis similar to *C*. *pseudotuberculosis* infections [5–8]. This variant, examined using genomics and proteomics, was initially named as the “wild boar cluster” (WBC) of *C*. *ulcerans* [5–7] and later reclassified as *C*. *silvaticum* [4].

The strain PO100/5 was isolated from caseous lymphadenitis lesions in a Black Alentejano pig (*Sus scrofa domesticus*) from a swine farm in the Alentejo region of Portugal. It was identified as *Corynebacterium pseudotuberculosis* by both biochemical tests (Api Coryne® kit) and by multiplex PCR and Pulsed Field Gel Electrophoresis [9]. Genome sequencing and *rpoB* phylogeny showed that this strain was closer to *C*. *ulcerans* and the genome was deposited in GenBank as a strain within this species (accession number CP021417.1). Recently the description of *C*. *silvaticum* was published and PO100/5 was suggested to be a strain of this species by *rpoB* phylogeny [4], while a genomic analysis of 28 *C*. *ulcerans* strains suggested that PO100/5, W25 and KL1196 could represent a new species [10]. KL1196 had already been classified as *C*. *silvaticum* [4].

Pigs and boars are reservoirs of *C*. *silvaticum* [4–7] and *C*. *ulcerans* [11–13], and are known to transmit pathogens to humans and other domestic animals [11, 12, 14]. Rapid, simple and reliable identification of this new species is essential for diagnosis, treatment and surveillance [15, 16]. To better understand the taxonomy of PO100/5, we performed a comparative analysis of 34 *C*. *silvaticum* and 80 *C*. *ulcerans* genomes, as well as other publicly available genomes from the *C*. *diphtheriae* group in order to explore the genomic diversity of *C*. *silvaticum* and to identify molecular markers of this species. We have reclassified PO100/5, established the other three strains recently deposited as *C*. *silvaticum*, and found both a unique sequence type and genes that can be useful for species classification.

## Materials and methods

### Genomes, assembly, and annotation

For the taxonomic, phylogenetic and genome plasticity analyses, a total of 120 genomes (S1 File) were selected, including 80 *C*. *ulcerans*, 34 *C*. *silvaticum* strains and six type strains from the *C*. *diphtheriae* group. Assembled genomes were retrieved from the Pathosystems Resource Integration Center (PATRIC) [17], while genomes available as sequencing reads were assembled in PATRIC using the SPAdes [18] strategy. All genomes were annotated using the Rapid Annotation using Subsystems Technology (RASTtk) pipeline [19] that is available in PATRIC.

### Taxonomic analysis

Average Nucleotide Identity (ANI) was estimated using FastANI v1.3 [20]. An automatic genome-based taxonomic analysis was performed using the Type (Strain) Genome Server (TYGS) (https://tygs.dsmz.de) [21]. This pipeline first identifies the closest type strains using MASH [22] for genomic sequences and BLAST [23] for 16S rRNA sequences. It then identifies the 10 closest type strains using Genome Blast Distance Phylogeny (GBDP) [24]. Finally, it clusters species and subspecies using digital DNA:DNA hybridization (dDDH) with a formula that is independent of genome length, being robust against the use of incomplete genomes (formula *d4*) [24]. It uses a threshold of 70 and 79%, respectively [25]. The difference in G+C content is also evaluated and expected to vary no more than 1% within a species [26]. Those analysis were performed for *C*. *ulcerans* strains from GenBank, using either the assembled genomes or sequencing data to check for misidentification of *C*. *silvaticum* strains.

Phylogenetic trees of the *rpoB* and *tox* genes were built using the Maximum Likelihood method [27] implemented in MEGA v10.1.6 [28]. The *tox* tree included all sequences from the genomes of *C*. *ulcerans* and included outgroups from *C*. *silvaticum* and *C*. *pseudotuberculosis*. *C*. *rouxii* was not included due to all sequenced genomes being *tox-* [2]. All trees were visualized using iTOL [29].

Multi Locus Sequence Typing (MLST) was performed using MLSTcheck [30], using the scheme for *C*. *diphtheriae* and *C*. *ulcerans* (genes *atpA*, *dnaE*, *dnaK*, *fusA*, *leuA*, *odhA* and *rpoB*) [13]. The Minimum Spanning Tree (MST) generated using goeBURST Full MSLT algorithm was built using PHYLOViZ v2.0 [31].

### Genome plasticity analysis

Prophages of PO100/5 were predicted using PHASTER [32]. Genomic islands were predicted using GIPSy v1.1.2 [33], with *C*. *ulcerans* NCTC7910^T^ and *C*. *pseudotuberculosis* ATCC19410^T^ used as references. A circular map was generated using BRIG v0.95 [34]. The presence of niche and virulence factors of *Corynebacterium* [35, 36] was verified using PATRIC’s Protein Family Sorter and Proteome Comparison tools. Gene neighborhoods were compared with other strains using the Artemis Comparison Tool 17.0.1 [37]. Signal peptide and conserved protein domains were verified using InterProScan [38], while cell wall sorting signal (CWSS) was verified using CW-PRED [39]. Mapping of sequencing reads to sequences of interest was performed using CLC Genomics Workbench v7 [40]. Specific nucleotide sequences in other genomes were identified using BLASTn [41] and GenBank non-redundant (nr) database [42].

The identification of groups of homologous genes (orthogroups) was performed using OrthoFinder v2.3.12 [43]. The output files Orthogroups.tsv and Orthogroups_UnassignedGenes.tsv from OrthoFinder were used as input for pangenome analyses using in-house scripts. The pangenome was represented by all orthogroups and the core genome by orthogroups conserved across all (100%) genomes. The accessory (or dispensable) genome was represented by the genes not conserved across all genomes. Within this subset, singletons were exclusive to a single genome, and shared genome (or dispensable genome minus singletons) are shared between two or more, but not all genomes [44]. To develop molecular markers, we identified subsets of orthogroups conserved and exclusive to a group of genomes (exclusive core). The development of a pangenome was calculated according to Heaps’ law fit formula *n* = κ*N^γ^, in which *n* is the number of genes, *N* is the number of genomes, and *κ* and γ (α = γ -1) are free parameters determined empirically. Heap’s law establishes the pangenome as being closed when α > 1 (γ < 0), which means that there is no significant increase with the addition of new genomes. It also defines a pangenome as open when α ≤ 1 (0 < γ < 1). The development of core genome and singletons were calculated using least-squares fit of the exponential regression decay n = κ*exp[-N/τ] + tg(θ), in which *n* is the number of genes, *N* is the number of genomes, and κ, τ, and tg(θ) are free parameters determined empirically [44]. A functional annotation of genes was performed using the eggNOG-mapper v2 [45].

The pathogenicity of *C*. *silvaticum* to humans was predicted using PathogenFinder v. 1.1 [46]. The prediction is performed using CD-HIT-2D [47] against a database of protein families associated with human pathogens. Strains PO100/5 and KL0182^T^ were used to represent livestock (domestic pig) and wild boars isolates, respectively.

## Results

### Taxonomic analysis

**Fig 1:**
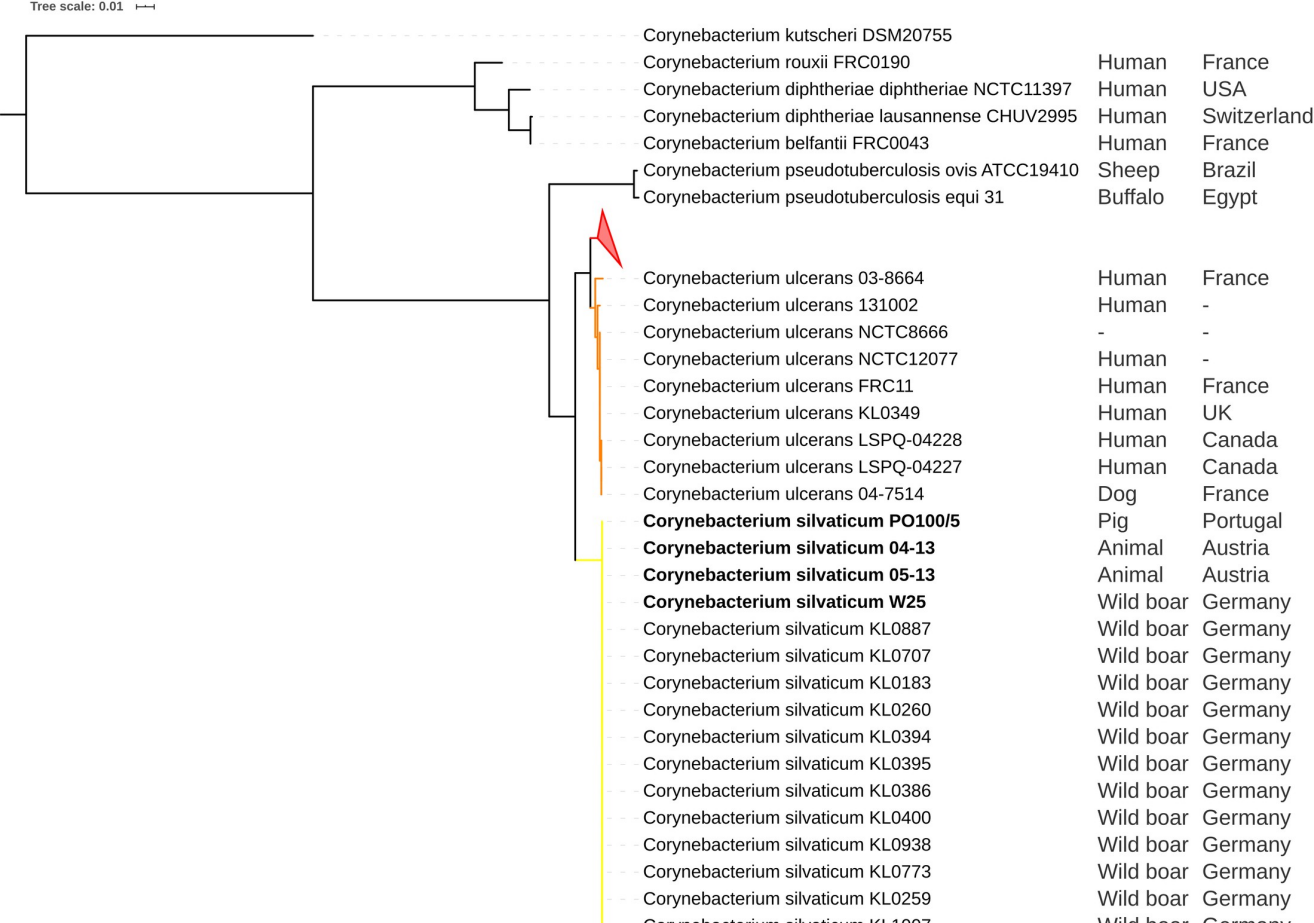
Phylogenetic tree of *rpoB* gene from *Corynebacterium* species. The phylogeny was inferred using the Maximum Likelihood method and the Tamura-Nei (TN93 + G) model implemented in the Mega v10.1.6. The *Corynebacterium ulcerans* strains PO100/5, 04–13, W25 and 05–13 cluster with the *Corynebacterium silvaticum* KL0182^T^ (yellow). *C*. *ulcerans* strains form lineage 1 (red, collapsed) and lineage 2 (orange).

**Fig 2:**
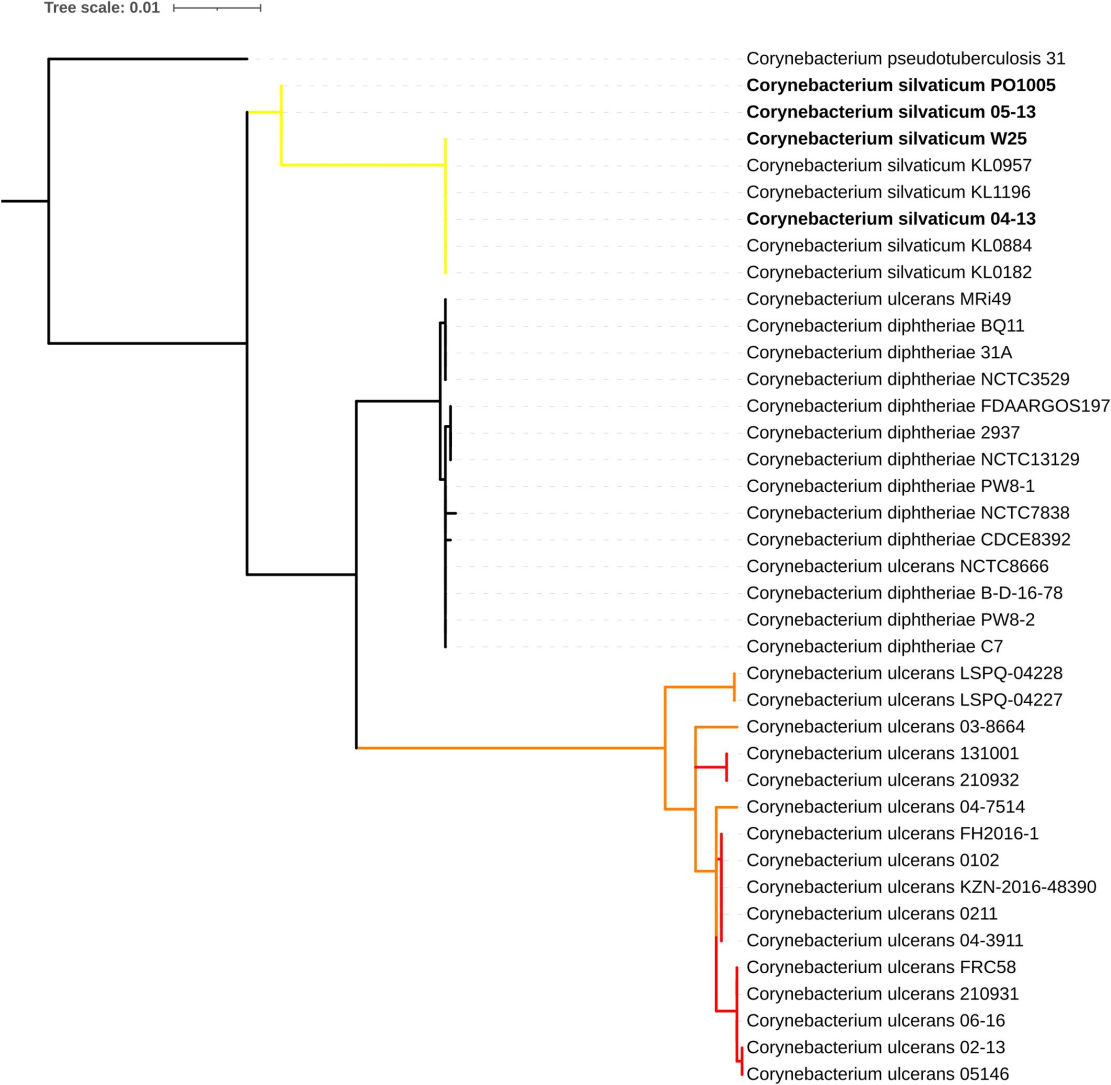
Phylogenetic tree of the *tox* gene from *Corynebacterium* species. The phylogeny was inferred using the Maximum Likelihood method and the Tamura-Nei (T92 + G) model implemented in Mega v10.1.6. The strains PO100/5, 04–13, W25 and 05–13 cluster with *Corynebacterium silvaticum*. Clade colors represent *rpoB* clades of *C*. *silvaticum* (yellow) and *C*. *ulcerans* (red and orange).

**Fig 3:**
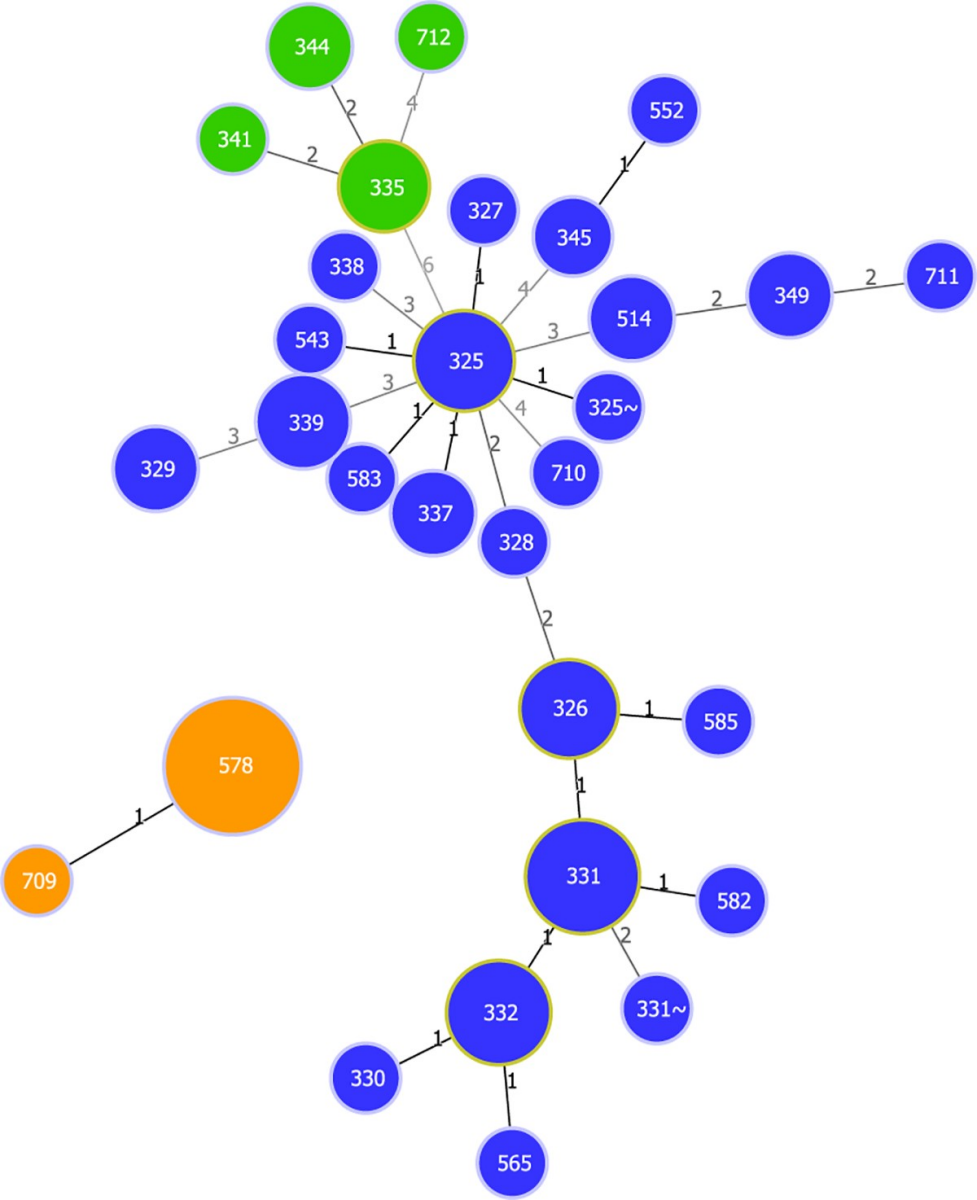
goeBURST diagram for the MLST data set of 38 *Corynebacterium silvaticum* and 76 *C*. *ulcerans* strains generated using PHILOViZ 2.0. Blue–*C*. *ulcerans* lineage 1. Green—*C*. *ulcerans* lineage 2. Orange–*C*. *silvaticum*. The numbers on the links indicate the number of divergent alleles between STs.

ANI results showed that *C*. *ulcerans* strains PO100/5, 04–13, 05–13 and W25 had identity values ≥ 99.74% with *C*. *silvaticum* KL0182^T^, and ≤ 91.03% with *C*. *ulcerans* NCTC7910^T^ (S2 File). The taxonomic classification using TYGS classified those strains as *C*. *silvaticum* due to genome and 16S rRNA GBDP trees, dDDh > 70% and G+C content difference > 1% with *C*. *ulcerans* genomes (S3 to S8 Files). In the *rpoB* phylogeny, the *C*. *ulcerans* strains PO100/5, 04–13, 05–13 and W25 clustered with *C*. *silvaticum* KL0182^T^, while other *C*. *ulcerans* strains formed two clades (Fig 1). In the phylogenetic tree of *tox* gene, the same four strains (PO100/5, 04–13, 05–13 and W25) also clustered with *C*. *silvaticum*, and were distinct from the *C*. *ulcerans*, *C*. *diphtheriae* and *C*. *pseudotuberculosis* clusters (Fig 2). MLST analysis classified strains 04–13, 05–13 and W25 as ST578 (53-60-121-70-76-66-57) and identified PO100/5 as having a unique and new sequence type, ST709 (53-60-121-70-76-82-57) where it differed from ST578 in the locus *odhA*. Due to those results those four strains were reclassified for the next analyses, changing the number of *C*. *silvaticum* genomes from 34 to 38 and *C*. *ulcerans* genomes from 80 to 76. Three new STs were identified, ST710 and ST711 in *C*. *ulcerans* lineage 1 and ST712 in lineage 2 (S9 File, Fig 3).

The taxonomic analyses led to additional insights. TYGS classified nine *C*. *ulcerans* strains (03–8664, 04–7514, 131002, FRC11, KL0349, LSPQ-04227, LSPQ-04228, NCTC8666 and NCTC12077) as being part of a potential new species. These genomes had dDDH values greater than 70% (99.8–75.9%) within them and less than 70% (63 to 67.2%) with other *C*. *ulcerans* genomes, although the difference in the G+C content was less than 1% (S3 File). In ANI analysis, those nine genomes were more similar to each other than to the other genomes. They had values between 95.52 and 96.57% with *C*. *ulcerans* NCTC7910^T^, and ≥ 97.82% when one of them (NCTC12077) was used as reference for the other eight (S2 File). MLST analysis classified them as having the unique sequence types ST335, ST341, ST344 and the new ST ST712 (Fig 3, S9 File). The ANI analysis showed 99.3% identity between *C*. *diphtheriae lausannense* strains CHUV2995 and *C*. *belfantii* FRC0043^T^ (S2 File). A further analysis using TYGS classified *C*. *diphtheriae lausannense* strains CHUV2995^T^ and CMCNS703 as belonging to *C*. *belfantii*, and as *C*. *diphtheriae* the non-type strains with genomes deposited in GenBank as *C*. *belfantii* (https://www.ncbi.nlm.nih.gov/genome/78252/) (S10 File). For this reason, *C*. *belfantii* 2937 was renamed to *C*. *diphtheriae* 2937 in Fig 2.

### Genome plasticity analysis

The presence of genes encoding 16 niche and virulence factors described in the genus *Corynebacterium* [35, 36] were examined in *C*. *silvaticum* (Table 1). The genes *rhuM*, *rpb* and *tspA* were not found, and all the pilus genes, except *spaB*, were found to be pseudogenized, lacking the signal peptide or CWSS. *C*. *silvaticum* has the two pilus gene clusters structured as *srtA*, *spaB*C, and *srtB*, *spaD*, *srtC* and *spaEF*, despite fragmentation of pilin genes. Only eight genomes had the *tox* gene (04–13, 05–13, KL0182, KL0884, KL0957, KL1196, PO100/5 and W25). PO100/5 and 05–13 do not have a two bases insertion (GG) after position 44, in a homopolymer of four guanines, that introduces a frameshift (S1 Fig). The mapping of PO100/5 sequencing reads to its assembled genome, showed an insertion of one guanine in the beginning of the homopolymer, in 5% of the reads (S2 Fig). The *tspA* gene was present in all *C*. *ulcerans* strains, but *rpb* was only found in strain 809, and *rhuM* was found in the 16 strains from Austria, France and Germany that had been isolated from humans, cats and dogs (02–13, FRC58, KL0195, KL0246-cb3, KL0251-cb4, KL0252-cb5, KL0349, KL0387-cb8, KL0475, KL0497, KL0541, KL0547, KL0796, KL0867, KL0880, NCTC12077).

**Table 1:** Presence of 16 known niche and virulence factors of *Corynebacterium* in *C*. *silvaticum*.

Gene	Product	Reference locus tag	Reference protein family	*C*. *silvaticum* protein family	Manual curation
*endoE*	Endoglycosidase E (former corynebacterial protease CP40)	CULC809_01974	PLF_1716_00006954	PLF_1716_00006954	Present
*cwlH*	Cell wall-associated hydrolase	CULC809_01521	PLF_1716_00062893	PLF_1716_00062893	Present
*nanH*	Sialidase (neuraminidase H)	CULC809_00434	PLF_1716_00002393	PLF_1716_00002393	Present
*pld*	Phospholipase D	CULC809_00040	PLF_1716_00029465	PLF_1716_00029465	Present
*rbp*	Shiga-like ribosome-binding protein	CULC809_00177	PLF_1716_00033486	-	Absent
*rhuM*	RhuM-like protein	CulFRC58_0285	PLF_1716_00026137	-	Absent
*rpfI*	Resuscitation-promoting factor-interacting protein	CULC809_01133	PLF_1716_00001449	PLF_1716_00001449	Present
*spaB*	Surface-anchored protein (minor pilus subunit)	CULC809_01980	PLF_1716_00010184	PLF_1716_00010184	Present
*spaC*	Surface-anchored protein (pilus tip protein)	CULC809_01979	PLF_1716_00004783	PLF_1716_00004783	Pseudogene, no cell wall sorting signal
*spaD*	Surface-anchored protein (major pilus subunit)	CULC809_01952	PLF_1716_00090862	PLF_1716_00102654	Pseudogene, no cell wall sorting signal
*spaE*	Surface-anchored protein (minor pilus subunit)	CULC809_01950	PLF_1716_00007274	PLF_1716_00079271	Pseudogene, no signal peptide
*spaF*	Surface-anchored protein (pilus tip protein)	CULC809_01949	PLF_1716_00006760	PLF_1716_00006760	Pseudogene, no signal peptide
*tox*	Diphtheria toxin	CULC0102_0213	PLF_1716_00005191	PLF_1716_00005191	Present in 8 out of 38 genomes
*tspA*	Trypsin-like serine protease	CULC809_01848	PLF_1716_00007827	-	Absent
*vsp1*	Venom serine protease	CULC809_00509	PLF_1716_00104602	PLF_1716_00104343	64% identity with *C*. *ulcerans* 809
*vsp2*	Venom serine protease	CULC809_01964	PLF_1716_00015799	PLF_1716_00116381	74% identity with *C*. *ulcerans* 809
*-*	*C*. *diphtheriae* DIP0733 homolog	CULC22_00609	PLF_1716_00030114	PLF_1716_00030114	Present

**Fig 4:**
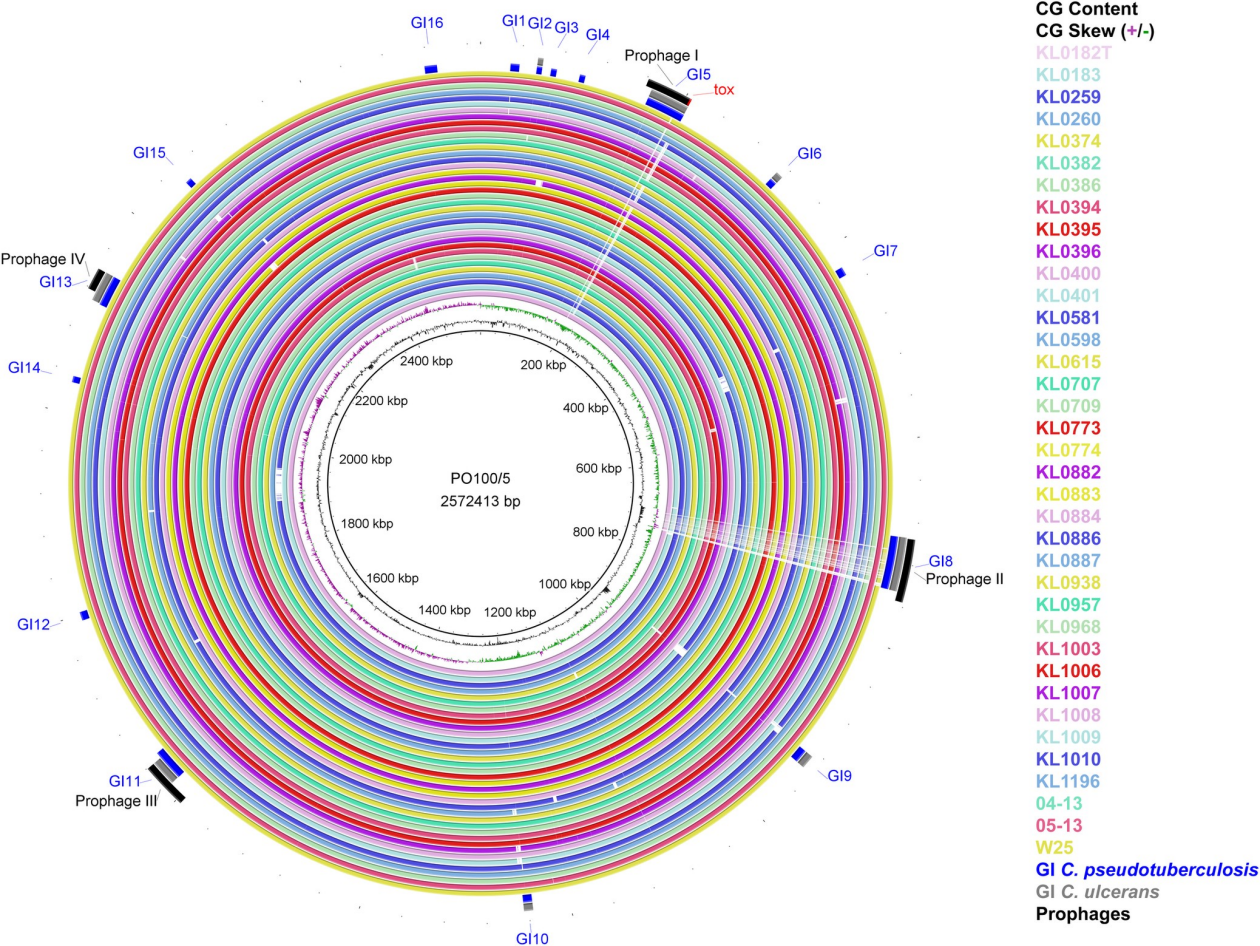
Circular map of *Corynebacterium silvaticum* genomes generated using BRIG v0.95. From inner to outer circle: strain PO100/5 (reference); CG content; CG Skew; strains KL0182^T^, KL0183, KL0259, KL0260, KL0374, KL0382, KL0386, KL0394, KL0395, KL0396, KL0400, KL0401, KL0581, KL0598, KL0615, KL0707, KL0709, KL0773, KL0774, KL0882, KL0883, KL0884, KL0886, KL0887, KL0938, KL0957, KL0968, KL1003, KL1006, KL1007, KL1008, KL1009, KL1010, KL1196, 04–13, 05–13, W25; genomic islands compared to *C*. *pseudotuberculosis* ATCC19410^T^ (blue) and *C*. *ulcerans* strain NCTC7910^T^ (grey); and prophages (black). Genomic islands (GI) and prophage detection were performed using GIPSy and PHASTER, respectively.

**Fig 5:**
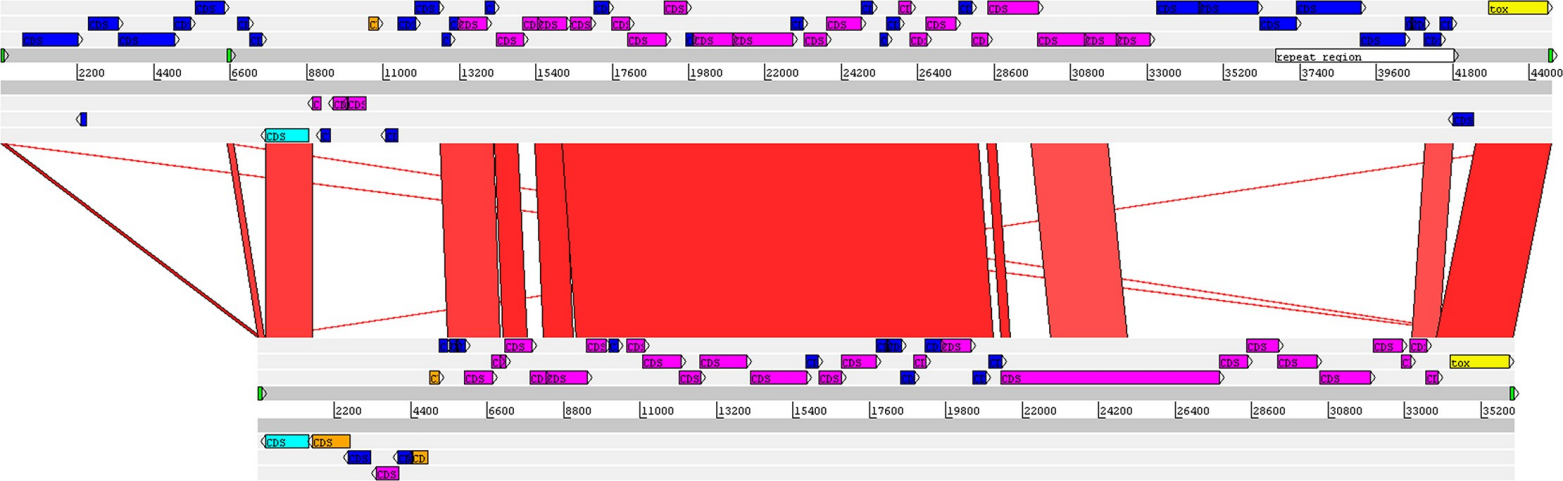
Alignment of *tox*^*+*^ prophages form *Corynebacterium silvaticum* PO100/5 and *C*. *ulcerans* 0102. The red lines connect sequences with at least 75% identity. Light blue–Phage integrase; Orange–Phage-related transcriptional regulator; Pink–Phage-related coding sequence (CDS); Yellow–Diphtheria toxin CDS; Dark blue–other CDS; Green–tRNA.

Sixteen and eight genomic islands were predicted by comparing PO100/5 with the reference strains *C*. *pseudotuberculosis* ATCC19410^T^ and *C*. *ulcerans* NCTC7910^T^, respectively. No island was detected when it was compared to *C*. *silvaticum* KL0182^T^ (Table 2, Fig 4). The genes in the discovered islands are provided in S11 File. They include one complete and three incomplete prophages. Prophage I harbors the *tox* gene and is similar to Gordonia phage Nyceirae (NC_031004.1) (S11 File, Fig 5). BLASTn of the *tox*^+^ prophage sequence using the GenBank nr database identified the best hits as *C*. *ulcerans* strains 0102 and 0211, with the same coverage (63%) and identity (92.68%). The best hits with other species were *C*. *diphtheriae lausannense* (*C*. *belfantii*) CMCNS703 (37 and 85.95%), *C*. *diphtheriae* strain B-D-16-78 (41 and 85.86%) and 15 strains of *C*. *pseudotuberculosis* (14 and 84.94%). Fig 5 shows the alignment of PO100/5 and *C*. *ulcerans* 0102 *tox*^*+*^ prophages.

**Table 2:** Genomic islands in strain PO100/5 compared to *Corynebacterium pseudotuberculosis* ATCC19410^T^ and *C*. *ulcerans* NCTC7910^T^.

n	Position compared to Cp	Size	Position compared to Cul	Size	Type	Prophage content
1	29362–38109	8.75 kb	-	-	-	-
2	55224–60448	5.22 kb	55224–60448	5.22 kb	PA	-
3	69256–74863	5.6 kb	-	-	PA, RE, SY	-
4	97842–103378	5.53 kb	-	-	PA, RE	-
5	167859–206438	38.58 kb	167859–206209	38.35 kb	-	Prophage I
6	311533–318567	7.03 kb	311533–318567	70.34 kb	-	-
7	422293–430839	85.46 kb	-	-	RE, SY	-
8	694806–746966	52.16 kb	694806–746966	52.16 kb	RE	Prophage II
9	925047–938225	13.18 kb	925047–938225	13.18 kb	-	-
10	1235769–1244625	8.86 kb	1235769–1244625	8.86 kb	-	-
11	1613625–1644953	31.33 kb	1614013–1639302	25.29 kb	-	Prophage III
12	1793740–1802246	8.5 kb	-	-	PA, ME	-
13	2029016–2035120	6.1 kb	-	-	-	-
14	2109299–2139505	30.2 kb	2110317–2139505	29.19 kb	-	Prophage IV
15	2255307–2261370	6.06 kb	-	-	-	-
16	2517632–2529863	12.23 kb	-	-	ME	-

Cp–*C*. *pseudotuberculosis*, Cul–*C*. *ulcerans*, PA–pathogenicity island, RE–resistance island, ME–metabolic island, SY–symbiotic island.

**Fig 6:**
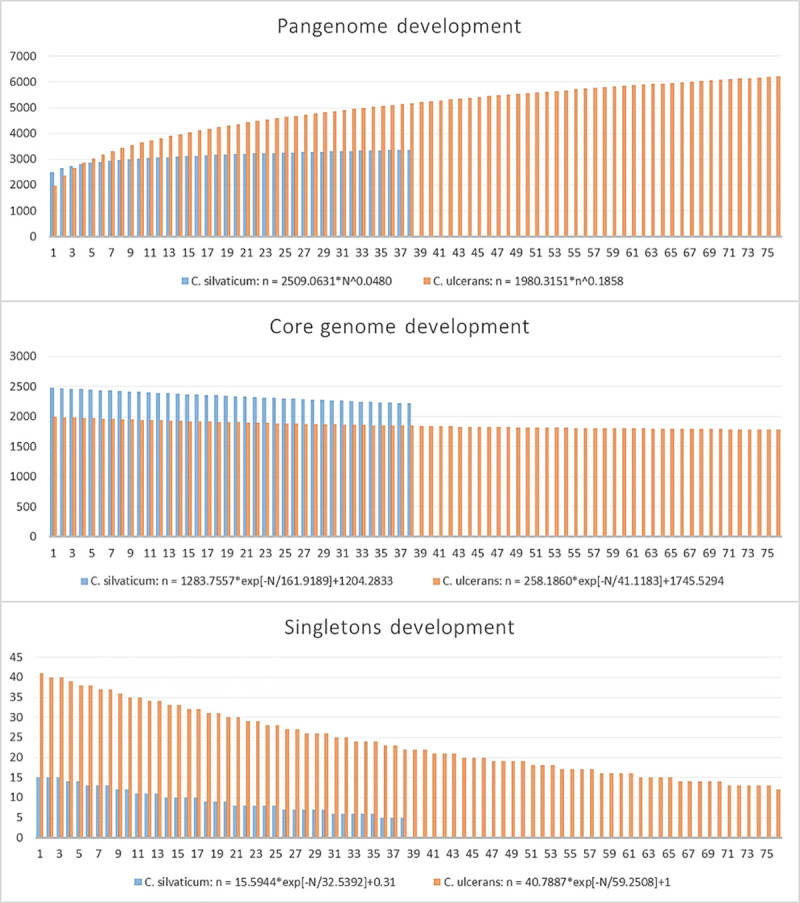
Pangenome, core genome and singletons development graphs and formulas for 38 genomes of *Corynebacterium silvaticum* and 76 *C*. *ulcerans* genomes.

**Fig 7:**
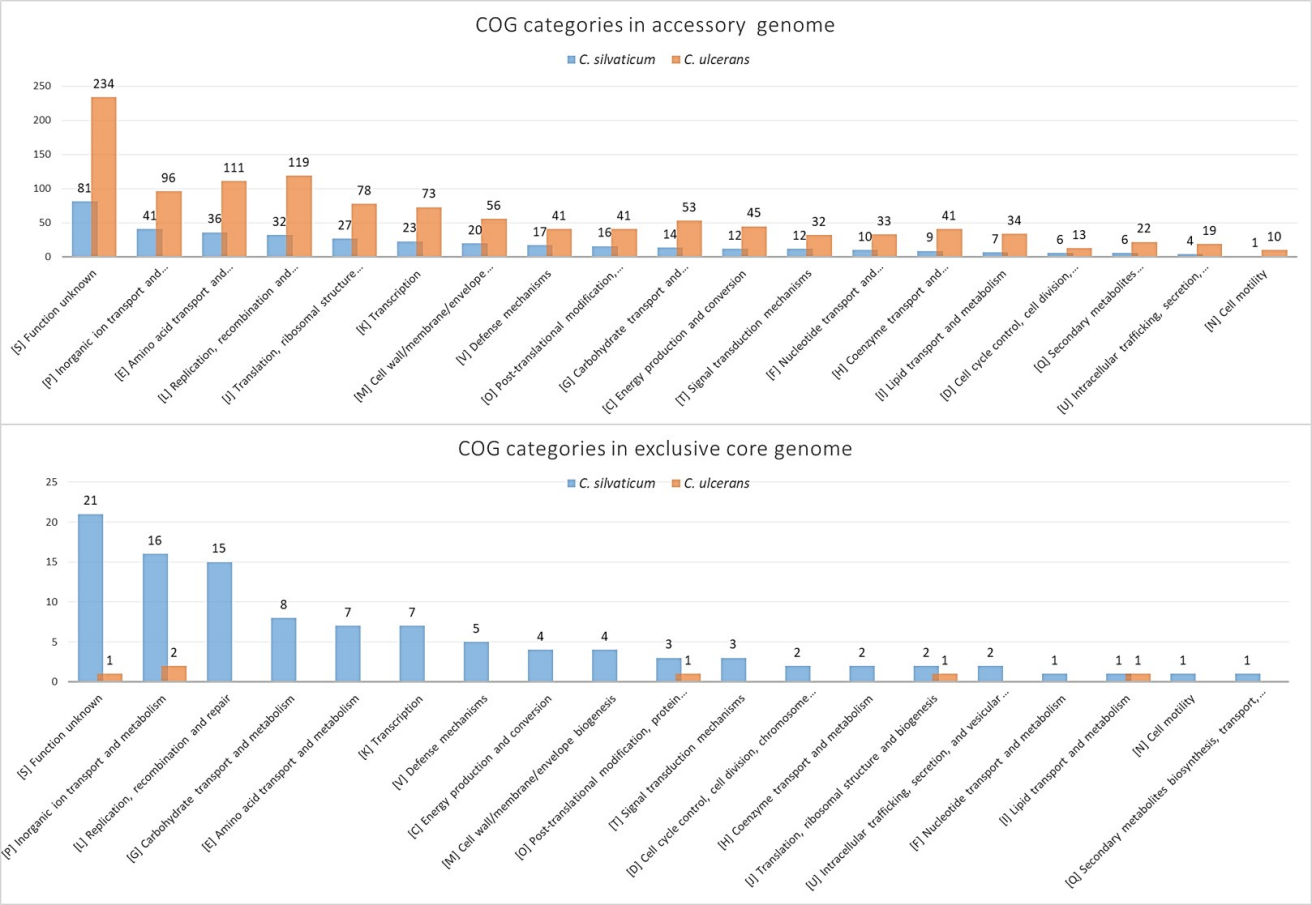
Clusters of orthologous groups (COGs) in the accessory and exclusive core genomes of *Corynebacterium silvaticum* and *C*. *ulcerans* annotated using eggNOG-mapper v2. COG categories are sorted from most abundant to less abundant in *C*. *silvaticum*. In *C*. *silvaticum*, 356 out of 793 proteins from the accessory genome and 93 out of 174 proteins of the exclusive core genome had a COG category. In *C*. *ulcerans*, 1,065 out of 2,064 proteins from the accessory genome and six out of nine proteins of the exclusive core genome had a COG category.

For the pangenome analysis, the number of orthogroups in each subset is shown in Table 3 and S12 File for *C*. *silvaticum* and *C*. *ulcerans*. The core genome represented 73.6% and 40% of orthogroups for *C*. *silvaticum* and *C*. *ulcerans*, respectively. The pangenome, core genome and singletons development graphs and formulas are shown in Fig 6. Both species had genes conserved in all strains that were absent in the other species, or the exclusive core. In *C*. *silvaticum*, 172 orthogroups were detected in this subset. They are represented in strain PO100/5 by 174 proteins, 81 of which are located across genomic islands 1, 2, 5, 6, 8, 9, 10, 11, 12 and 14. *C*. *ulcerans* lineage 2 had a hypothetical protein with 37 amino acids (S12 File). A graph comparing the distribution of Cluster of Homologous Groups (COG) categories of the exclusive core genome of both species is shown in Fig 7.

**Table 3:** Number of genes (orthogroups) in subsets across *Corynebacterium silvaticum* and *C*. *ulcerans* genomes.

Species	Genomes	Pangenome	Core genome	Accessory genome	Shared genome	Singletons	α
*C*. *silvaticum*	38	3,002	2,209	703	603	190	0.9520
*C*. *ulcerans*	76	4,351	1,747	2,604	1,706	898	0.8142
*C*. *silvaticum* and *C*. *ulcerans*	114	4,916	1,618	3,298	2,349	949	_

The pangenome is the entire repertoire of orthogroups, the core genome is the subset conserved across all genomes (100%), the accessory genome is the subset not conserved across all genomes, singletons are exclusive from a genome, and the shared genome are orthogroups shared by two or more, but not all genomes.

Finally, PO100/5 (isolated from domestic pig) and KL0182^T^ (wild boar) were predicted to be potential human pathogens by PathogenFinder, with 14 and 13 matches with proteins associated to pathogens, respectively (S13 File).

## Discussion

Strain PO100/5 was originally classified as *C*. *pseudotuberculosis*. Its resistance profile was tested for 13 antimicrobial compounds (Amoxycillin/Clavulanic acid, Ampicillin, Chloramphenicol, Cephalexin, Gentamicin, Cefotaxime, Enrofloxacin, Nalidixic acid, Penicillin G, Streptomycin, Sulfamethoxazole/Trimethoprim, Tetracycline and Vancomycin) and it was found to be resistant to nalidixic acid and streptomycin [9]. It was suggested as *C*. *silvaticum* by a recent *rpoB* phylogeny [4]. We analyzed the genome diversity of this species, using publicly available genomes from the *C*. *diphtheriae* group (S1 File).

Taxonomic analysis showed that PO100/5, W25, 04–13 and 05–13 are strains of the recently described *C*. *silvaticum* [4]. This is supported by ANI values above 95% [20] (S2 File), genome and 16S rRNA GBDP clustering [24], dDDH > 70%, G+C content difference > 1% with *C*. *ulcerans* genomes [21, 24, 26] (S3–S5 Files), *rpoB* phylogenetic clustering (Fig 1) and the unique sequence type ST578 from *C*. *silvaticum* [4] (Fig 3). Strain PO100/5 has the new ST709 (S9 File, Fig 4). The misclassification of those strains is expected as, prior to the development of methods to identify *C*. *silvaticum* [4, 5], the use of biochemistry tests (API Coryne and VITEK2-compact) and the clinical picture would classify these strains as *C*. *pseudotuberculosis* [4, 48], while DNA sequence analysis and Fourier-transform Infrared Spectroscopy would classify it as *C*. *ulcerans* [6, 7, 48].

Analysis of genome plasticity identified unique characteristics *C*. *silvaticum*. The analysis of 16 known niche and virulence factors showed the absence of *rpb*, *rhuM*, *and tsA*. *spaB* was the only non-fragmented pili gene in *C*. *silvaticum* (Table 1). The Shiga-like ribosome-binding protein (*rpb*) has a ribosome inactivating protein domain that has only been reported in *C*. *ulcerans* 809 [35, 49]. The new species also has a RhuM-like protein (*rhuM*), which has only been seen previously in the *C*. *ulcerans* strain KL0387 [50]. A RhuM mutant of *Salmonella enterica* had a significant decrease in epithelial cell invasion [51]. We identified this protein in 15 other strains from humans, dogs and cats form Austria, France and Germany.

Serine proteases can promote the survival and dissemination of pathogens in the host [52], and we looked for these virulence factors in the genomes we analyzed (Table 1). Venom serine proteases (*vsp1* and *vsp2*) and Trypsin-like serine protease (*tspA*) are secreted proteases that could have multiple potential pathogenic functions [53]. There is homology between the two serine proteases found in *C*. *ulcerans* in this new species (Table 1), but *tspA* was not found in *C*. *silvaticum*. Its absence could be used as a marker to differentiate it from *C*. *ulcerans*.

Bacterial pili are adhesion structures required for colonization of host tissues. The *Corynebacterium* pili are SpaA-type, with a heterotrimeric structure composed by major (pilus shaft), minor and tip pilins, the last two required for adhesion. The pilus is assembled and anchored to the cell wall by the housekeeping sortase SrtA and pili sortases SrtB and SrtC [54]. As seen in *C*. *ulcerans* [35], *C*. *silvaticum* has the two pili gene clusters *spaB*C and *spaDEF*, although only *spaB* appears to be functional due to the presence of a signal peptide and a CWSS. The SpaB is a minor pilin that in *C*. *diphtheriae* has a role in adhesion on pharyngeal epithelial cells and could be functional when linked to the cell wall [55] as shown for the heterodimeric structure SpaB-SpaC in *C*. *diphtheriae* [56] and suggested for *C*. *ulcerans* [35].

The *tox* gene was found in only eight out of the 38 *C*. *silvaticum* strains (04–13, 05–13, KL0182, KL0884, KL0957, KL1196, PO100/5 and W25), although the strains lacking it were reported to be NTTB [6]. This can be seen in the circular map as a blank space in the *tox* gene region of the other 30 strains (Fig 4). The absence of the *tox* gene in the other strains could be the result of an assembly artifact, due to a repetitive region prior to this gene. Additionally, the *tox* sequences from PO100/5 and 05–13 lack the insertion of two guanines in position 44 (S1 and S2 Figs) that causes pseudogenization, characteristic of other *C*. *silvaticum* strains [10]. A recent publication showed that strains 04–13 and 05–13 from Austria produce the *tox* transcript by reverse transcriptase quantitative PCR (RT-qPCR) [8]. As 04–13 has the frameshift, 05–13 and PO10/5 could be the only known toxigenic *C*. *silvaticum* strains. The production of DT has yet to be tested.

In PO100/5, four incomplete prophages were found, one harboring the *tox* gene. When PO100/5 was compared to *C*. *pseudotuberculosis* ATCC 19410^T^, sixteen genomic islands were identified. When it was compared to *C*. *ulcerans* NCTC7910^T^, only eight islands were found. Four of the islands contained the prophages: GI5, GI8, GI11 and GI13 (S11 File, Figs 4 and 5). No island was found in comparison to *C*. *silvaticum* KL0182^T^. Genomic islands are mobile genetic elements (MGEs) acquired by horizontal gene transfer that can provide adaptive traits [33]. In a previous study, MGEs containing *tox* in *C*. *diphtheriae* were identified as known prophages, while in *C*. *ulcerans* they can be different prophages or an alternative pathogenicity island. These mobile elements showed nearly species-specific clades, including the atypical *C*. *ulcerans* clade that now represents *C*. *silvaticum*. This implies independent events of acquisition of virulence factors in zoonotic species that could influence their pathogenic potential [6].

*C*. *silvaticum* was estimated to be more genetically homogeneous than *C*. *ulcerans* and to have a pangenome near to being closed, with bigger values of core genome development (Fig 6) and α closer to 1 (Table 3). This result could be influenced by the samples of *C*. *silvaticum* being from only two separate countries, Germany (n = 37) and Portugal (n = 1), and from two different species of host (*Sus scrofa* and *Capreolus capreolus*). This estimation could change once more genomes are sequenced. A total of 172 and 8 orthogroups were uniquely shared by all *C*. *silvaticum* and *C*. *ulcerans*, respectively, some in the described genomic islands (S12 File, Fig 7). For *C*. *silvaticum*, the most abundant functions are involved in nutrient acquisition such as transport and metabolism of inorganic ions, carbohydrates and amino acids (COG categories E, G and P), or are related to phages or immunity against them (COG category L). For example, two of them are a Type I restriction-modification system [57] in genomic island 11 and an “ABC-type dipeptide oligopeptide nickel transport system”. The function of those genes in the phenotype and infection must be investigated, but they are candidates for genetic markers for a rapid and cost-effective diagnostic using multiplex polymerase chain reaction (PCR) [58–60] and other established methods [4, 5].

In addition to being of veterinary importance, *C*. *silvaticum* could have medical relevance, as strains PO100/5 and KL1082 were predicted to be potential human pathogens (S13 File). The known host range of *C*. *silvaticum* is limited to wild boars, domestic pigs and roe deer [4, 7, 9]. Wild boars are reservoirs for viruses, bacteria and other parasites that can be transmitted to livestock and humans, during opportunities provided by deforestation and use of lands for agricultural purposes, hunting activities and consumption of wild boar meat [14]. Although they are transmitted additionally by other hosts, pigs and boars are a reservoir of *C*. *ulcerans*, which can cause zoonotic transmission to humans [11–13]. By the same route, *C*. *silvaticum* could be transmitted to humans and cause infection. In addition, it could be misidentified as *C*. *ulcerans* or *C*. *pseudotuberculosis* due to limitations in the standard methodology [4, 5].

Additionally, the TYGS results suggest that nine *C*. *ulcerans* corresponding to lineage 2 [49] is a potential new species, with dDDH of less than 70% with lineage 1 genomes. (S3–S6 Files). Further investigation is required to verify whether this lineage could be classified as a new species. Recently, *C*. *belfantii* and *C*. *diphtheriae lausannense* were suggested as synonyms [2]. Our analysis using TYGS corroborated that suggestion. In addition, besides strains FRC0043^T^, CHUV2995^T^ and CMCNS703, the other nine genomes deposited in GenBank as *C*. *belfantii* (https://www.ncbi.nlm.nih.gov/genome/78252/) were classified as *C*. *diphtheriae* (S10 File). These results suggest the limitation of using only one cutoff as a parameter for taxonomic classification.

## Conclusions

The taxonomic analysis shows PO100/5 and four other genomes deposited as *C*. *ulcerans* are from the recently described species *C*. *silvaticum*. The comparative genomic analysis showed this species is more genetically homogeneous than *C*. *ulcerans*, has SpaB as the only probably functional pilin subunit, and has conserved genomic islands and 172 genes that could be used as molecular markers for PCR identification. In contrast to the other strains from the same species, PO100/5 is the first one to be isolated from livestock and outside Germany and Austria, and to have the unique ST709. A non pseudogenized *tox* gene in PO100/5 and 05–13 suggest those strains could produce the diphtheria toxin.

## Supporting information

S1 Fig Alignment of tox gene from *Corynebacterium silvaticum*, *C. ulcerans*, *C. pseudotuberculosis* and *C. ulcerans*. The alignment was performed using MUSCLE algorithm implemented in MEGA v10.1.6. *C*. *silvaticum* strains PO100/5 and 05–13 do not have a two guanines insertion that lead to a frameshift in other strains from this species.
(TIF)

S2 Fig Mapping of sequencing reads to the tox gene of *Corynebacterium silvaticum* strain PO100/5. (TIF)

S1 File Genomes of *Corynebacterium* species used for taxonomic analysis of the PO100/5 strain. (XLSX)

S2 File Average Nucleotide Identity and digital DNA-DNA hybridization among strains of *Corynebacterium*. Strains highlighted in blue are *Corynebacterium ulcerans* strains from lineage 2. ANI values under 78.64% are showed as "NA".
(XLSX)

S3 File Taxonomic classification of 80 *Corynebacterium ulcerans* genomes from GenBank by digital DNA-DNA hybridization and G+C content. The analysis was performed using Type Strain Genome Server.
(XLSX)

S4 File Taxonomic classification of 20 *Corynebacterium ulcerans* genomes from GenBank. The analysis was performed using Type Strain Genome Server. Strains 04/13 and 05/13 were classified as *C*. *silvaticum*, while 03–8664, 04–7514, 131002 were classified as a potential new species.
(PDF)

S5 File Taxonomic classification of 15 *Corynebacterium ulcerans* genomes from GenBank. The analysis was performed using Type Strain Genome Server. Strains PO100/5 and W25 were classified as *C*. *silvaticum*, while FRC11, LSPQ-04227, LSPQ-04228, NCTC8666 and NCTC12077 were classified as a potential new species.
(PDF)

S6 File Taxonomic classification of 20 *Corynebacterium ulcerans* genomes from GenBank, available as sequencing data. The analysis was performed using Type Strain Genome Server. Strain KL0349 was classified as a potential new species.
(PDF)

S7 File Taxonomic classification of 20 *Corynebacterium ulcerans* genomes from GenBank, available as sequencing data. The analysis was performed using Type Strain Genome Server. All strains were classified as *C*. *ulcerans*.
(PDF)

S8 File Taxonomic classification of five *Corynebacterium ulcerans* genomes from GenBank, available as sequencing data. The analysis was performed using Type Strain Genome Server. All strains were classified as *C*. *ulcerans*.
(PDF)

S9 File Taxonomic classification of 2 *Corynebacterium diphtheriae lausannense* and 10 *C. belfantii* genomes from GenBank. The analysis was performed using Type Strain Genome Server. *C*. *diphtheriae lausannense* strains CHUV2995^T^ and CMCNS703 were classified as *C*. *belfantii*, while *C*. *belfantii* strains except the type strain FRC0043^T^ were classified as *C*. *diphtheriae*.
(XLSX)

S10 File Multilocus sequence typing data of *Corynebacterium silvaticum* and *C. ulcerans* genomes. The analysis was performed using MLSTchecker.
(PDF)

S11 File Genomic islands content in strain PO100/5. (XLSX)

S12 File Pangenome analysis of 38 *Corynebacterium silvaticum* and 76 *C. ulcerans* samples. Gene homology groups were predicted using OrthoFinder v2.12.2 and functional annotation was performed using eggNOG-mapper v2.
(XLSX)

S13 File Probability of pathogenicity for humans. PathogenFinder v. 1.1 was used to identify proteins associated to bacterial pathogens in the proteome of *C*. *silvaticum* PO100/5 (isolated from domestic pig) and KL0182^T^ (wild boar).
(XLSX)
