# Supplementary figures and images for: Taxonomic classification of strain PO100/5 shows a broader geographic distribution and genetic markers of the recently described Corynebacterium silvaticum
Source: PLoS One. 2020 Dec 21;15(12):e0244210. doi: 10.1371/journal.pone.0244210 (PMC7751848; doi:10.1371/journal.pone.0244210)

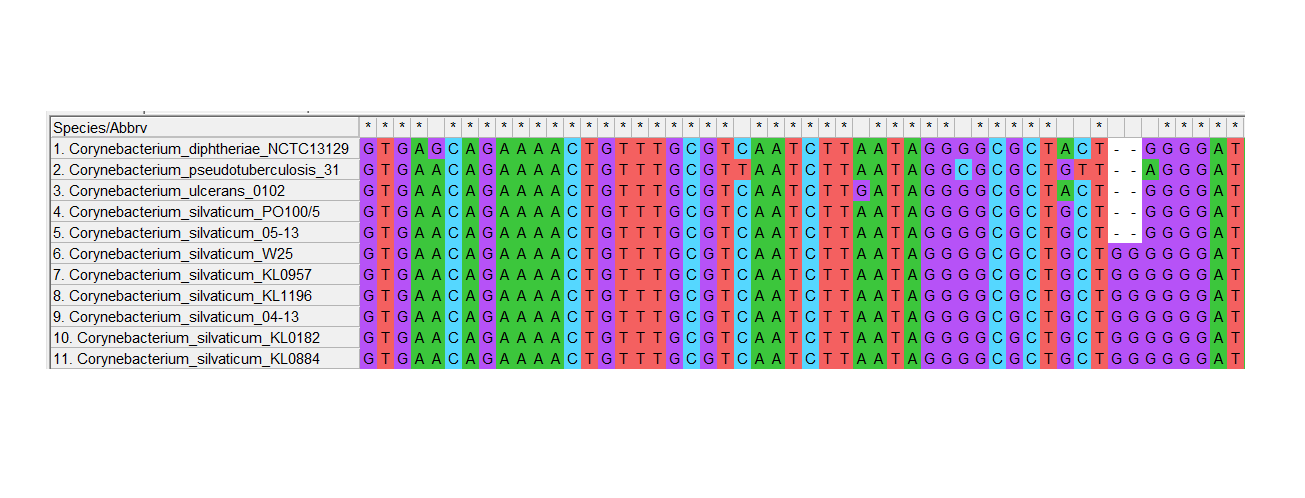

Supplement: S1 Fig — The alignment was performed using MUSCLE algorithm implemented in MEGA v10.1.6. C. silvaticum strains PO100/5 and 05–13 do not have a two guanines insertion that lead to a frameshift in other strains from this species. (TIF) [file pone.0244210.s001.tif]

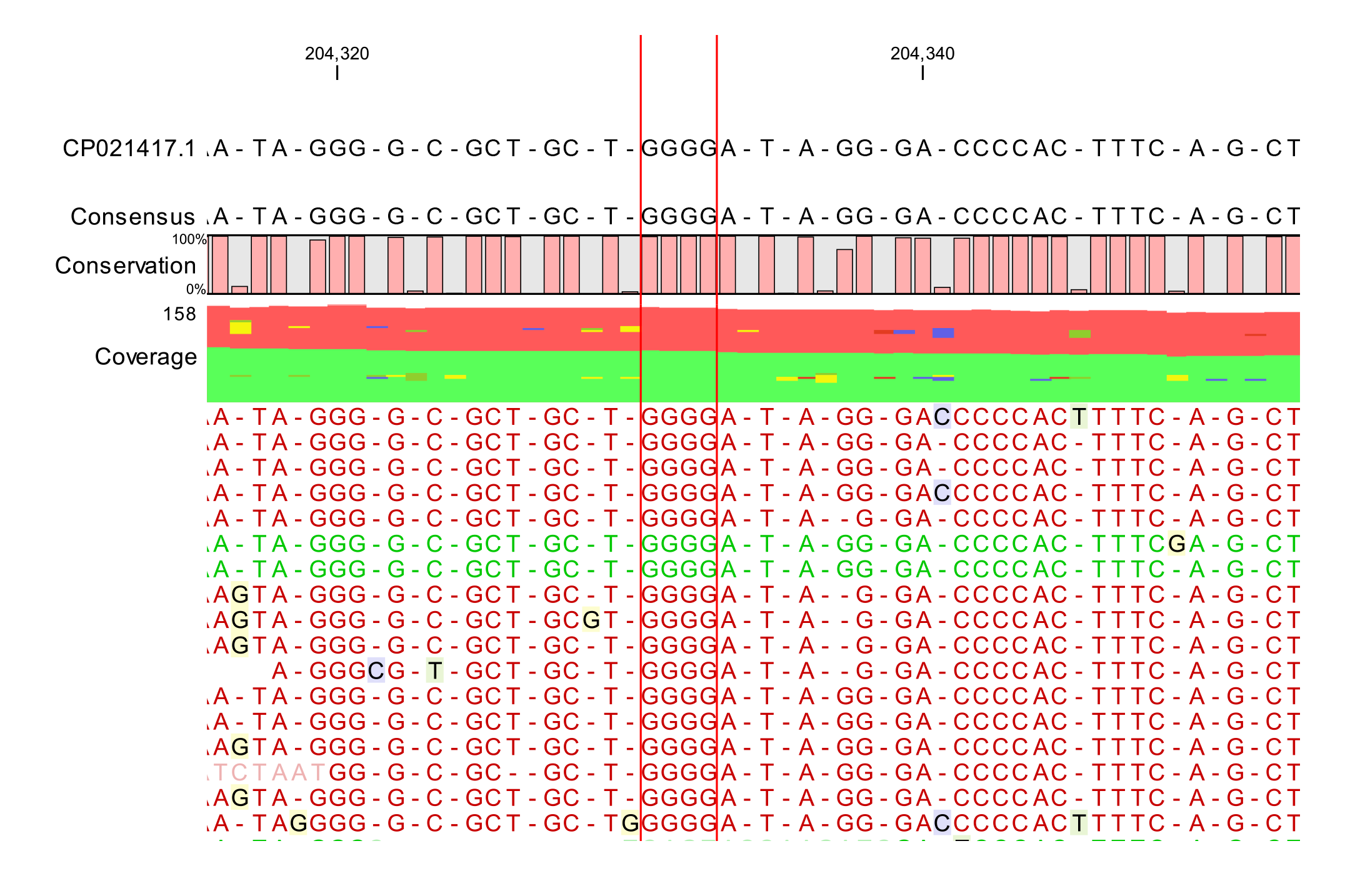

Supplement: S2 Fig — (TIF) [file pone.0244210.s002.tif]
