# Supplementary material for: Taxonomic classification of strain PO100/5 shows a broader geographic distribution and genetic markers of the recently described Corynebacterium silvaticum
Source: PLoS One. 2020 Dec 21;15(12):e0244210. doi: 10.1371/journal.pone.0244210 (PMC7751848; doi:10.1371/journal.pone.0244210)
Supplement: S7 File — The analysis was performed using Type Strain Genome Server. All strains were classified as C. ulcerans. (PDF) [file pone.0244210.s009.pdf]

TYPE (STRAIN) GENOME SERVER

JOB SUMMARY

PRINT DATE: 2020-06-11 22:42:34 +0200

JOB ID: 3f1a8641-39bd-4f4c-b3ac-0c6455711329

RESULT PAGE: https://tygs.dsmz.de/user_results/show?guid=3f1a8641-39bd-4f4c-b3ac-0c6455711329

Table 1: Phylogenies

Publication-ready versions of both the genome-scale GBDP tree and the 16S rRNA gene sequence tree can be customized and exported either in SVG (vector graphic) or PNG format from within the phylogeny viewers in your TYGS result page. For publications the **SVG format is recommended** because it is lossless, always keeps its high resolution and can also be easily converted to other popular formats such as PDF or EPS. Please follow the link provided above!

Table 2: Identification

The below list contains the result of the TYGS species identification routine.

Explanation of remarks that might occur in the below table:

remark [R1]: The TYGS type strain database is automatically updated on an almost daily basis. However, if a particular type strain genome is not available in the TYGS database, this can have several reasons which are detailed in the FAQ. You can request an extended 16S rRNA gene analysis via the 16S tree viewer found in your result page to detect **not yet genome-sequenced** type strains relevant for your study.

remark [R2]: > 70% dDDH value (formula d_4) and (almost) minimal dDDH values for gene-content formulae d_0 and d_6 indicate a potentially unreliable identification result and should thus be checked via the 16S rRNA gene sequence similarity. Such strong deviations can, in principle, be caused by sequence contamination.

remark [R3]: G+C content difference of > 1 % indicates a potentially unreliable identification result because within species G+C content varies no more than 1 %, if computed from genome sequences (PMID: 24505073).

Strain	Conclusion	Identification result	Remark
**'KL0475'**	belongs to known species	*Corynebacterium ulcerans*	
**'KL0483'**	belongs to known species	*Corynebacterium ulcerans*	
**'KL0497'**	belongs to known species	*Corynebacterium ulcerans*	
**'KL0501'**	belongs to known species	*Corynebacterium ulcerans*	
**'KL0515'**	belongs to known species	*Corynebacterium ulcerans*	
**'KL0540'**	belongs to known species	*Corynebacterium ulcerans*	
**'KL0541'**	belongs to known species	*Corynebacterium ulcerans*	
**'KL0547'**	belongs to known species	*Corynebacterium ulcerans*	
**'KL0556'**	belongs to known species	*Corynebacterium ulcerans*	
**'KL0785'**	belongs to known species	*Corynebacterium ulcerans*	
**'KL0796'**	belongs to known species	*Corynebacterium ulcerans*	
**'KL0818'**	belongs to known species	*Corynebacterium ulcerans*	
**'KL0825'**	belongs to known species	*Corynebacterium ulcerans*	
**'KL0832'**	belongs to known species	*Corynebacterium ulcerans*	
**'KL0840'**	belongs to known species	*Corynebacterium ulcerans*	

Strain	Conclusion	Identification result	Remark
**'KL0846'**	belongs to known species	*Corynebacterium ulcerans*	
**'KL0853'**	belongs to known species	*Corynebacterium ulcerans*	
**'KL0867'**	belongs to known species	*Corynebacterium ulcerans*	
**'KL0870'**	belongs to known species	*Corynebacterium ulcerans*	
**'KL0876'**	belongs to known species	*Corynebacterium ulcerans*	

Table 3: Pairwise comparisons of user genomes vs. type-strain genomes

The following table contains the pairwise dDDH values between your user genomes and the selected type-strain genomes. The dDDH values are provided along with their confidence intervals (C.I.) for the three different GBDP formulas:

- formula d_0 (a.k.a. GGDC formula 1): length of all HSPs divided by total genome length
- formula d_4 (a.k.a. GGDC formula 2): sum of all identities found in HSPs divided by overall HSP length
- formula d_6 (a.k.a. GGDC formula 3): sum of all identities found in HSPs divided by total genome length

Note: Formula d_4 is independent of genome length and is thus robust against the use of incomplete draft genomes. For other reasons for preferring formula d_4, see the FAQ.

Query	Subject	d_0	C.I. d_0	d_4	C.I. d_4	d_6	C.I. d_6	Diff. G+C Percent
**'KL0785'**	**'KL0818'**	100.0	[100.0 - 100.0]	100.0	[100.0 - 100.0]	100.0	[100.0 - 100.0]	0.01
**'KL0515'**	**'KL0785'**	99.4	[98.8 - 99.7]	99.9	[99.8 - 100.0]	99.7	[99.5 - 99.9]	0.04
**'KL0515'**	**'KL0818'**	99.3	[98.7 - 99.6]	99.9	[99.8 - 100.0]	99.7	[99.4 - 99.8]	0.03
**'KL0483'**	**'KL0846'**	98.2	[97.0 - 98.9]	99.6	[99.4 - 99.8]	99.1	[98.6 - 99.5]	0.08
**'KL0825'**	**'KL0846'**	99.6	[99.1 - 99.8]	99.6	[99.4 - 99.8]	99.8	[99.6 - 99.9]	0.0
**'KL0475'**	**'KL0846'**	95.2	[93.0 - 96.8]	99.6	[99.3 - 99.8]	97.4	[96.1 - 98.3]	0.01
**'KL0483'**	**'KL0832'**	98.4	[97.3 - 99.1]	99.6	[99.3 - 99.7]	99.2	[98.7 - 99.6]	0.09
**'KL0825'**	**'KL0840'**	99.6	[99.3 - 99.8]	99.6	[99.3 - 99.8]	99.8	[99.7 - 99.9]	0.0
**'KL0840'**	**'KL0846'**	100.0	[99.9 - 100.0]	99.6	[99.3 - 99.8]	100.0	[99.9 - 100.0]	0.0
**'KL0540'**	**'KL0547'**	97.9	[96.6 - 98.8]	99.6	[99.3 - 99.7]	99.0	[98.3 - 99.4]	0.02
**'KL0475'**	**'KL0832'**	95.5	[93.4 - 97.0]	99.5	[99.2 - 99.7]	97.6	[96.4 - 98.4]	0.01
**'KL0475'**	**'KL0840'**	95.6	[93.5 - 97.1]	99.5	[99.2 - 99.7]	97.7	[96.4 - 98.5]	0.01
**'KL0547'**	**'KL0867'**	99.7	[99.4 - 99.9]	99.5	[99.3 - 99.7]	99.9	[99.7 - 99.9]	0.0
**'KL0796'**	**'KL0846'**	97.7	[96.2 - 98.6]	99.5	[99.2 - 99.7]	98.8	[98.1 - 99.3]	0.04
**'KL0483'**	**'KL0840'**	98.4	[97.2 - 99.0]	99.5	[99.2 - 99.7]	99.2	[98.6 - 99.5]	0.08
**'KL0796'**	**'KL0832'**	97.9	[96.5 - 98.7]	99.5	[99.2 - 99.7]	98.9	[98.3 - 99.4]	0.04
**'KL0540'**	**'KL0867'**	98.7	[97.7 - 99.2]	99.5	[99.2 - 99.7]	99.4	[98.9 - 99.6]	0.02
**'KL0832'**	**'KL0846'**	100.0	[99.9 - 100.0]	99.5	[99.2 - 99.7]	100.0	[100.0 - 100.0]	0.01
**'KL0825'**	**'KL0832'**	99.7	[99.3 - 99.8]	99.5	[99.2 - 99.7]	99.9	[99.7 - 99.9]	0.0
**'KL0796'**	**'KL0840'**	97.8	[96.4 - 98.7]	99.5	[99.3 - 99.7]	98.9	[98.2 - 99.3]	0.04
**'KL0832'**	**'KL0840'**	100.0	[100.0 - 100.0]	99.5	[99.2 - 99.7]	100.0	[100.0 - 100.0]	0.0
**'KL0483'**	**'KL0825'**	98.2	[96.9 - 98.9]	99.4	[99.0 - 99.6]	99.1	[98.5 - 99.5]	0.08

Query	Subject	d_0	C.I. d_0	d_4	C.I. d_4	d_6	C.I. d_6	Diff. G+C Percent
**'KL0796'**	**'KL0825'**	97.9	[96.5 - 98.7]	99.4	[99.0 - 99.6]	98.9	[98.2 - 99.4]	0.04
**'KL0475'**	**'KL0796'**	97.0	[95.3 - 98.1]	99.3	[99.0 - 99.6]	98.5	[97.6 - 99.0]	0.03
**'KL0475'**	**'KL0825'**	96.4	[94.5 - 97.6]	99.2	[98.7 - 99.5]	98.0	[97.0 - 98.7]	0.01
**'KL0483'**	**'KL0796'**	98.9	[98.0 - 99.4]	98.9	[98.3 - 99.3]	99.4	[99.0 - 99.7]	0.05
**'KL0475'**	**'KL0483'**	96.3	[94.4 - 97.6]	98.8	[98.1 - 99.2]	98.0	[96.9 - 98.7]	0.08
**'KL0541'**	**'KL0867'**	97.3	[95.7 - 98.3]	98.5	[97.8 - 99.0]	98.5	[97.7 - 99.1]	0.03
**'KL0541'**	**'KL0547'**	97.8	[96.5 - 98.7]	98.5	[97.7 - 99.0]	98.8	[98.1 - 99.3]	0.02
**'KL0540'**	**'KL0541'**	97.0	[95.3 - 98.1]	98.4	[97.6 - 98.9]	98.3	[97.4 - 98.9]	0.01
**'KL0541'**	**'KL0840'**	95.4	[93.2 - 96.9]	97.0	[95.9 - 97.9]	97.2	[95.9 - 98.1]	0.05
**'KL0541'**	**'KL0846'**	95.2	[93.0 - 96.8]	97.0	[95.9 - 97.9]	97.1	[95.7 - 98.0]	0.05
**'KL0846'**	**'KL0867'**	98.4	[97.3 - 99.1]	97.0	[95.8 - 97.8]	99.0	[98.4 - 99.4]	0.02
**'KL0541'**	**'KL0832'**	95.5	[93.3 - 97.0]	97.0	[95.9 - 97.9]	97.3	[96.0 - 98.2]	0.06
**'KL0840'**	**'KL0867'**	98.5	[97.5 - 99.2]	96.9	[95.7 - 97.8]	99.1	[98.5 - 99.5]	0.03
**'KL0832'**	**'KL0867'**	98.6	[97.6 - 99.2]	96.9	[95.7 - 97.8]	99.2	[98.6 - 99.5]	0.03
**'KL0547'**	**'KL0846'**	97.7	[96.2 - 98.6]	96.9	[95.7 - 97.7]	98.6	[97.8 - 99.1]	0.03
**'KL0547'**	**'KL0832'**	97.9	[96.6 - 98.7]	96.8	[95.6 - 97.7]	98.7	[98.0 - 99.2]	0.03
**'KL0547'**	**'KL0840'**	97.8	[96.5 - 98.7]	96.8	[95.6 - 97.7]	98.7	[97.9 - 99.2]	0.03
**'KL0540'**	**'KL0846'**	99.3	[98.7 - 99.6]	96.8	[95.6 - 97.7]	99.6	[99.2 - 99.8]	0.04
**'KL0483'**	**'KL0867'**	98.2	[96.9 - 98.9]	96.7	[95.5 - 97.6]	98.9	[98.2 - 99.3]	0.06
**'KL0825'**	**'KL0867'**	98.3	[97.1 - 99.0]	96.7	[95.4 - 97.6]	98.9	[98.3 - 99.4]	0.03
**'KL0541'**	**'KL0825'**	95.5	[93.3 - 97.0]	96.7	[95.4 - 97.6]	97.2	[95.9 - 98.2]	0.05
**'KL0483'**	**'KL0547'**	97.4	[95.9 - 98.4]	96.7	[95.5 - 97.6]	98.4	[97.5 - 99.0]	0.05
**'KL0497'**	**'KL0547'**	98.4	[97.3 - 99.1]	96.6	[95.3 - 97.5]	99.0	[98.4 - 99.4]	0.04
**'KL0547'**	**'KL0825'**	97.5	[96.0 - 98.5]	96.6	[95.3 - 97.5]	98.5	[97.6 - 99.1]	0.03
**'KL0540'**	**'KL0840'**	99.4	[98.8 - 99.7]	96.6	[95.3 - 97.5]	99.6	[99.3 - 99.8]	0.05
**'KL0547'**	**'KL0796'**	97.1	[95.5 - 98.2]	96.6	[95.4 - 97.5]	98.3	[97.3 - 98.9]	0.01
**'KL0483'**	**'KL0540'**	98.6	[97.6 - 99.2]	96.6	[95.4 - 97.5]	99.1	[98.5 - 99.5]	0.04

Query	Subject	d_0	C.I. d_0	d_4	C.I. d_4	d_6	C.I. d_6	Diff. G+C Percent
**'KL0497'**	**'KL0867'**	98.9	[98.0 - 99.4]	96.5	[95.3 - 97.5]	99.3	[98.8 - 99.6]	0.03
**'KL0796'**	**'KL0867'**	97.7	[96.3 - 98.6]	96.5	[95.3 - 97.5]	98.6	[97.7 - 99.1]	0.01
**'KL0540'**	**'KL0832'**	99.5	[98.9 - 99.7]	96.5	[95.2 - 97.5]	99.6	[99.3 - 99.8]	0.05
**'KL0540'**	**'KL0825'**	98.8	[97.9 - 99.3]	96.4	[95.1 - 97.4]	99.2	[98.7 - 99.6]	0.05
**'KL0497'**	**'KL0540'**	98.8	[97.9 - 99.3]	96.4	[95.1 - 97.4]	99.2	[98.7 - 99.6]	0.06
**'KL0475'**	**'KL0867'**	95.6	[93.5 - 97.1]	96.3	[95.0 - 97.3]	97.3	[95.9 - 98.2]	0.02
**'KL0501'**	**'KL0540'**	99.1	[98.4 - 99.5]	96.3	[95.0 - 97.3]	99.4	[99.0 - 99.7]	0.05
**'KL0540'**	**'KL0796'**	97.4	[95.9 - 98.4]	96.3	[94.9 - 97.3]	98.4	[97.5 - 99.0]	0.01
**'KL0475'**	**'KL0547'**	94.9	[92.5 - 96.5]	96.3	[95.0 - 97.3]	96.8	[95.3 - 97.8]	0.02
**'KL0501'**	**'KL0867'**	98.4	[97.3 - 99.1]	96.3	[94.9 - 97.3]	99.0	[98.3 - 99.4]	0.03
**'KL0497'**	**'KL0541'**	96.2	[94.2 - 97.5]	96.2	[94.8 - 97.2]	97.6	[96.4 - 98.4]	0.06
**'KL0501'**	**'KL0547'**	97.7	[96.2 - 98.6]	96.2	[94.9 - 97.2]	98.5	[97.7 - 99.1]	0.03
**'KL0475'**	**'KL0541'**	94.4	[91.9 - 96.1]	96.2	[94.8 - 97.2]	96.4	[94.9 - 97.5]	0.04
**'KL0541'**	**'KL0796'**	98.7	[97.8 - 99.3]	96.2	[94.9 - 97.2]	99.2	[98.6 - 99.5]	0.01
**'KL0475'**	**'KL0540'**	94.1	[91.6 - 95.9]	96.0	[94.6 - 97.1]	96.3	[94.6 - 97.4]	0.04
**'KL0483'**	**'KL0541'**	97.1	[95.4 - 98.1]	95.7	[94.3 - 96.8]	98.1	[97.1 - 98.8]	0.03
**'KL0497'**	**'KL0796'**	98.2	[96.9 - 98.9]	95.6	[94.1 - 96.7]	98.8	[98.0 - 99.3]	0.05
**'KL0497'**	**'KL0840'**	99.6	[99.1 - 99.8]	95.5	[94.0 - 96.6]	99.6	[99.3 - 99.8]	0.01
**'KL0497'**	**'KL0846'**	99.5	[99.0 - 99.7]	95.4	[93.9 - 96.6]	99.6	[99.2 - 99.8]	0.01
**'KL0497'**	**'KL0825'**	98.9	[98.1 - 99.4]	95.4	[93.9 - 96.6]	99.2	[98.7 - 99.6]	0.01
**'KL0497'**	**'KL0832'**	99.6	[99.2 - 99.8]	95.4	[93.9 - 96.5]	99.7	[99.4 - 99.8]	0.0
**'KL0501'**	**'KL0541'**	95.2	[93.0 - 96.8]	95.4	[93.9 - 96.6]	96.9	[95.5 - 97.9]	0.06
**'KL0483'**	**'KL0497'**	97.4	[95.8 - 98.4]	95.4	[93.8 - 96.5]	98.3	[97.3 - 98.9]	0.09
**'KL0501'**	**'KL0846'**	99.6	[99.1 - 99.8]	95.3	[93.8 - 96.5]	99.6	[99.3 - 99.8]	0.01
**'KL0475'**	**'KL0497'**	95.9	[93.9 - 97.3]	95.3	[93.7 - 96.4]	97.4	[96.1 - 98.2]	0.02
**'KL0501'**	**'KL0825'**	99.1	[98.3 - 99.5]	95.2	[93.6 - 96.4]	99.3	[98.8 - 99.6]	0.0
**'KL0501'**	**'KL0796'**	96.8	[95.0 - 98.0]	95.2	[93.6 - 96.4]	97.9	[96.8 - 98.6]	0.04

Query	Subject	d_0	C.I. d_0	d_4	C.I. d_4	d_6	C.I. d_6	Diff. G+C Percent
**'KL0501'**	**'KL0832'**	99.7	[99.4 - 99.9]	95.2	[93.7 - 96.4]	99.7	[99.5 - 99.8]	0.0
**'KL0501'**	**'KL0840'**	99.7	[99.3 - 99.8]	95.2	[93.7 - 96.4]	99.7	[99.4 - 99.8]	0.0
**'KL0483'**	**'KL0501'**	97.5	[95.9 - 98.4]	95.2	[93.7 - 96.4]	98.3	[97.4 - 98.9]	0.09
**'KL0475'**	**'KL0501'**	94.2	[91.7 - 96.0]	94.9	[93.3 - 96.2]	96.2	[94.5 - 97.3]	0.01
**'KL0497'**	**'KL0501'**	99.1	[98.4 - 99.5]	94.3	[92.6 - 95.6]	99.3	[98.8 - 99.6]	0.0
**'KL0853'**	**'KL0876'**	96.9	[95.2 - 98.0]	93.1	[91.2 - 94.7]	97.8	[96.6 - 98.6]	0.13
**'KL0870'**	**'KL0876'**	96.2	[94.2 - 97.5]	91.9	[89.8 - 93.6]	97.2	[95.9 - 98.1]	0.09
**'KL0515'**	**'KL0853'**	96.5	[94.7 - 97.8]	90.9	[88.7 - 92.7]	97.3	[96.0 - 98.2]	0.08
**'KL0853'**	**'KL0870'**	96.5	[94.7 - 97.8]	90.6	[88.3 - 92.4]	97.3	[96.0 - 98.2]	0.04
**'KL0497'**	**'KL0870'**	96.5	[94.6 - 97.7]	90.6	[88.3 - 92.4]	97.3	[96.0 - 98.2]	0.06
**'KL0785'**	**'KL0853'**	97.6	[96.1 - 98.5]	90.4	[88.1 - 92.2]	98.0	[96.9 - 98.7]	0.04
**'KL0818'**	**'KL0853'**	97.6	[96.1 - 98.5]	90.3	[88.1 - 92.2]	98.0	[96.9 - 98.7]	0.05
**'KL0497'**	**'KL0876'**	98.0	[96.7 - 98.8]	89.7	[87.4 - 91.7]	98.3	[97.3 - 98.9]	0.03
**'KL0846'**	**'KL0870'**	96.5	[94.6 - 97.7]	89.4	[87.0 - 91.4]	97.2	[95.8 - 98.1]	0.05
**'KL0475'**	**'KL0870'**	94.9	[92.6 - 96.5]	89.4	[87.0 - 91.4]	96.1	[94.4 - 97.2]	0.05
**'KL0840'**	**'KL0870'**	96.6	[94.8 - 97.8]	89.3	[86.9 - 91.3]	97.3	[95.9 - 98.2]	0.06
**'KL0832'**	**'KL0870'**	96.8	[95.0 - 97.9]	89.3	[86.9 - 91.3]	97.3	[96.0 - 98.2]	0.06
**'KL0825'**	**'KL0870'**	97.8	[96.5 - 98.7]	89.2	[86.8 - 91.2]	98.1	[97.0 - 98.8]	0.06
**'KL0846'**	**'KL0876'**	98.2	[96.9 - 98.9]	89.1	[86.7 - 91.1]	98.3	[97.3 - 98.9]	0.04
**'KL0818'**	**'KL0876'**	97.1	[95.4 - 98.2]	89.1	[86.7 - 91.1]	97.6	[96.3 - 98.4]	0.08
**'KL0501'**	**'KL0870'**	96.6	[94.8 - 97.8]	89.1	[86.7 - 91.1]	97.2	[95.9 - 98.1]	0.06
**'KL0785'**	*Corynebacterium ulcerans* NCTC 7910	97.0	[95.3 - 98.1]	89.1	[86.7 - 91.2]	97.5	[96.3 - 98.3]	0.05
**'KL0785'**	**'KL0876'**	97.1	[95.4 - 98.2]	89.1	[86.7 - 91.1]	97.6	[96.3 - 98.4]	0.09
**'KL0515'**	*Corynebacterium ulcerans* NCTC 7910	98.0	[96.8 - 98.8]	89.1	[86.7 - 91.1]	98.2	[97.2 - 98.9]	0.01
**'KL0475'**	**'KL0876'**	91.5	[88.4 - 93.8]	89.1	[86.7 - 91.1]	93.5	[91.3 - 95.2]	0.05
**'KL0818'**	*Corynebacterium ulcerans* NCTC 7910	97.0	[95.3 - 98.1]	89.1	[86.7 - 91.1]	97.5	[96.3 - 98.3]	0.04
**'KL0832'**	**'KL0876'**	98.4	[97.3 - 99.1]	89.0	[86.6 - 91.0]	98.4	[97.5 - 99.0]	0.03

Query	Subject	d_0	C.I. d_0	d_4	C.I. d_4	d_6	C.I. d_6	Diff. G+C Percent
**'KL0796'**	**'KL0876'**	94.5	[92.1 - 96.2]	89.0	[86.6 - 91.1]	95.7	[94.0 - 97.0]	0.08
**'KL0515'**	**'KL0876'**	98.1	[96.8 - 98.9]	89.0	[86.6 - 91.1]	98.3	[97.3 - 98.9]	0.05
**'KL0825'**	**'KL0876'**	97.3	[95.8 - 98.3]	89.0	[86.6 - 91.0]	97.7	[96.5 - 98.5]	0.04
**'KL0483'**	**'KL0876'**	95.3	[93.1 - 96.8]	89.0	[86.6 - 91.0]	96.3	[94.7 - 97.4]	0.12
**'KL0840'**	**'KL0876'**	98.3	[97.1 - 99.0]	89.0	[86.6 - 91.0]	98.4	[97.5 - 99.0]	0.04
**'KL0541'**	*Corynebacterium ulcerans* NCTC 7910	93.3	[90.6 - 95.2]	88.9	[86.5 - 91.0]	94.8	[92.9 - 96.3]	0.05
**'KL0796'**	**'KL0870'**	97.2	[95.6 - 98.3]	88.9	[86.5 - 90.9]	97.6	[96.4 - 98.4]	0.02
**'KL0867'**	*Corynebacterium ulcerans* NCTC 7910	96.8	[95.0 - 97.9]	88.9	[86.5 - 91.0]	97.3	[96.0 - 98.2]	0.03
**'KL0547'**	*Corynebacterium ulcerans* NCTC 7910	95.9	[93.8 - 97.3]	88.9	[86.5 - 91.0]	96.7	[95.2 - 97.7]	0.03
**'KL0547'**	**'KL0876'**	95.2	[93.0 - 96.8]	88.8	[86.3 - 90.8]	96.2	[94.6 - 97.4]	0.07
**'KL0541'**	**'KL0876'**	92.6	[89.7 - 94.7]	88.8	[86.4 - 90.9]	94.3	[92.2 - 95.8]	0.09
**'KL0497'**	**'KL0853'**	96.0	[94.0 - 97.4]	88.7	[86.2 - 90.7]	96.8	[95.3 - 97.8]	0.1
**'KL0867'**	**'KL0876'**	96.2	[94.3 - 97.5]	88.7	[86.3 - 90.8]	96.9	[95.5 - 97.9]	0.06
**'KL0540'**	*Corynebacterium ulcerans* NCTC 7910	97.9	[96.6 - 98.8]	88.7	[86.3 - 90.8]	98.1	[97.1 - 98.8]	0.05
**'KL0540'**	**'KL0876'**	97.4	[95.9 - 98.4]	88.6	[86.2 - 90.7]	97.7	[96.6 - 98.5]	0.08
**'KL0497'**	*Corynebacterium ulcerans* NCTC 7910	97.8	[96.4 - 98.6]	88.6	[86.1 - 90.6]	98.0	[96.9 - 98.7]	0.01
**'KL0876'**	*Corynebacterium ulcerans* NCTC 7910	97.7	[96.2 - 98.6]	88.5	[86.0 - 90.6]	97.9	[96.8 - 98.6]	0.04
**'KL0853'**	*Corynebacterium ulcerans* NCTC 7910	95.3	[93.1 - 96.8]	88.5	[86.1 - 90.6]	96.2	[94.6 - 97.4]	0.1
**'KL0483'**	**'KL0870'**	97.4	[95.9 - 98.4]	88.4	[86.0 - 90.5]	97.7	[96.5 - 98.5]	0.03
**'KL0475'**	*Corynebacterium ulcerans* NCTC 7910	91.2	[88.1 - 93.5]	88.4	[85.9 - 90.5]	93.2	[90.9 - 95.0]	0.01
**'KL0515'**	**'KL0870'**	94.8	[92.5 - 96.5]	88.4	[86.0 - 90.5]	95.9	[94.2 - 97.1]	0.04
**'KL0840'**	*Corynebacterium ulcerans* NCTC 7910	98.1	[96.9 - 98.9]	88.3	[85.8 - 90.4]	98.2	[97.2 - 98.8]	0.0
**'KL0846'**	*Corynebacterium ulcerans* NCTC 7910	98.0	[96.7 - 98.8]	88.3	[85.8 - 90.4]	98.1	[97.1 - 98.8]	0.0
**'KL0796'**	*Corynebacterium ulcerans* NCTC 7910	94.3	[91.8 - 96.0]	88.2	[85.7 - 90.3]	95.4	[93.6 - 96.8]	0.04
**'KL0825'**	*Corynebacterium ulcerans* NCTC 7910	97.2	[95.5 - 98.2]	88.2	[85.7 - 90.3]	97.5	[96.3 - 98.3]	0.0
**'KL0832'**	*Corynebacterium ulcerans* NCTC 7910	98.2	[97.0 - 98.9]	88.2	[85.7 - 90.3]	98.3	[97.3 - 98.9]	0.0
**'KL0541'**	**'KL0870'**	95.3	[93.1 - 96.8]	88.2	[85.7 - 90.3]	96.2	[94.5 - 97.3]	0.0

Query	Subject	d_0	C.I. d_0	d_4	C.I. d_4	d_6	C.I. d_6	Diff. G+C Percent
**'KL0870'**	*Corynebacterium ulcerans* NCTC 7910	94.7	[92.3 - 96.3]	88.1	[85.6 - 90.2]	95.7	[94.0 - 97.0]	0.06
**'KL0483'**	*Corynebacterium ulcerans* NCTC 7910	95.2	[92.9 - 96.7]	88.0	[85.5 - 90.1]	96.1	[94.4 - 97.3]	0.08
**'KL0501'**	**'KL0876'**	97.8	[96.3 - 98.6]	88.0	[85.5 - 90.1]	97.9	[96.8 - 98.6]	0.03
**'KL0785'**	**'KL0870'**	96.2	[94.2 - 97.5]	87.9	[85.4 - 90.1]	96.8	[95.3 - 97.8]	0.0
**'KL0818'**	**'KL0870'**	96.3	[94.3 - 97.6]	87.9	[85.4 - 90.0]	96.8	[95.4 - 97.9]	0.01
**'KL0846'**	**'KL0853'**	96.2	[94.3 - 97.5]	87.9	[85.3 - 90.0]	96.8	[95.3 - 97.8]	0.09
**'KL0840'**	**'KL0853'**	96.4	[94.5 - 97.6]	87.9	[85.4 - 90.0]	96.9	[95.5 - 97.9]	0.1
**'KL0556'**	*Corynebacterium ulcerans* NCTC 7910	98.1	[96.9 - 98.9]	87.9	[85.3 - 90.0]	98.2	[97.2 - 98.8]	0.01
**'KL0832'**	**'KL0853'**	96.5	[94.6 - 97.7]	87.8	[85.3 - 89.9]	97.0	[95.6 - 98.0]	0.1
**'KL0501'**	**'KL0853'**	96.4	[94.4 - 97.6]	87.8	[85.2 - 89.9]	96.9	[95.5 - 97.9]	0.1
**'KL0501'**	*Corynebacterium ulcerans* NCTC 7910	97.9	[96.6 - 98.7]	87.7	[85.2 - 89.9]	98.0	[96.9 - 98.7]	0.0
**'KL0825'**	**'KL0853'**	95.3	[93.1 - 96.9]	87.7	[85.2 - 89.9]	96.2	[94.5 - 97.3]	0.1
**'KL0497'**	**'KL0818'**	96.8	[95.0 - 98.0]	87.6	[85.0 - 89.7]	97.2	[95.9 - 98.1]	0.05
**'KL0497'**	**'KL0785'**	96.7	[94.9 - 97.9]	87.6	[85.0 - 89.7]	97.1	[95.8 - 98.1]	0.06
**'KL0556'**	**'KL0870'**	95.4	[93.2 - 96.9]	87.6	[85.0 - 89.7]	96.2	[94.5 - 97.3]	0.05
**'KL0497'**	**'KL0515'**	97.8	[96.4 - 98.7]	87.4	[84.8 - 89.6]	97.9	[96.8 - 98.6]	0.02
**'KL0475'**	**'KL0853'**	92.6	[89.8 - 94.7]	87.3	[84.8 - 89.5]	94.1	[92.0 - 95.7]	0.09
**'KL0540'**	**'KL0853'**	95.7	[93.6 - 97.2]	87.3	[84.7 - 89.5]	96.4	[94.8 - 97.5]	0.05
**'KL0556'**	**'KL0876'**	97.6	[96.1 - 98.5]	87.2	[84.6 - 89.4]	97.7	[96.5 - 98.5]	0.05
**'KL0867'**	**'KL0870'**	94.8	[92.5 - 96.5]	87.2	[84.6 - 89.4]	95.7	[94.0 - 97.0]	0.03
**'KL0547'**	**'KL0870'**	94.0	[91.4 - 95.8]	87.2	[84.6 - 89.4]	95.1	[93.2 - 96.5]	0.03
**'KL0853'**	**'KL0867'**	95.5	[93.3 - 97.0]	87.2	[84.6 - 89.4]	96.2	[94.6 - 97.4]	0.07
**'KL0541'**	**'KL0853'**	94.0	[91.5 - 95.8]	87.2	[84.6 - 89.4]	95.2	[93.3 - 96.5]	0.04
**'KL0818'**	**'KL0832'**	97.2	[95.6 - 98.3]	87.1	[84.5 - 89.3]	97.4	[96.2 - 98.3]	0.05
**'KL0785'**	**'KL0840'**	97.0	[95.3 - 98.1]	87.1	[84.5 - 89.3]	97.3	[96.0 - 98.2]	0.05
**'KL0515'**	**'KL0846'**	97.9	[96.5 - 98.7]	87.1	[84.5 - 89.3]	97.9	[96.8 - 98.6]	0.01
**'KL0796'**	**'KL0853'**	95.5	[93.4 - 97.0]	87.1	[84.5 - 89.3]	96.2	[94.6 - 97.4]	0.06

Query	Subject	d_0	C.I. d_0	d_4	C.I. d_4	d_6	C.I. d_6	Diff. G+C Percent
**'KL0785'**	**'KL0832'**	97.1	[95.5 - 98.2]	87.1	[84.5 - 89.3]	97.4	[96.1 - 98.2]	0.05
**'KL0515'**	**'KL0840'**	98.0	[96.7 - 98.8]	87.1	[84.5 - 89.3]	98.0	[97.0 - 98.7]	0.01
**'KL0818'**	**'KL0840'**	97.1	[95.4 - 98.2]	87.1	[84.5 - 89.3]	97.4	[96.1 - 98.2]	0.05
**'KL0785'**	**'KL0846'**	96.8	[95.1 - 98.0]	87.1	[84.6 - 89.3]	97.2	[95.8 - 98.1]	0.05
**'KL0818'**	**'KL0846'**	96.9	[95.2 - 98.1]	87.1	[84.6 - 89.3]	97.3	[95.9 - 98.2]	0.04
**'KL0515'**	**'KL0541'**	92.9	[90.1 - 94.9]	87.0	[84.4 - 89.2]	94.3	[92.2 - 95.8]	0.04
**'KL0483'**	**'KL0515'**	94.9	[92.6 - 96.6]	87.0	[84.4 - 89.2]	95.8	[94.1 - 97.0]	0.07
**'KL0541'**	**'KL0556'**	94.1	[91.6 - 95.9]	87.0	[84.4 - 89.2]	95.2	[93.3 - 96.6]	0.04
**'KL0515'**	**'KL0832'**	98.1	[96.9 - 98.9]	87.0	[84.4 - 89.2]	98.1	[97.1 - 98.8]	0.02
**'KL0475'**	**'KL0556'**	92.3	[89.4 - 94.5]	87.0	[84.4 - 89.2]	93.9	[91.7 - 95.5]	0.0
**'KL0515'**	**'KL0825'**	97.0	[95.3 - 98.1]	87.0	[84.4 - 89.2]	97.3	[96.0 - 98.2]	0.01
**'KL0540'**	**'KL0818'**	96.7	[94.8 - 97.9]	86.9	[84.3 - 89.1]	97.0	[95.6 - 98.0]	0.0
**'KL0540'**	**'KL0785'**	96.6	[94.7 - 97.8]	86.9	[84.3 - 89.1]	96.9	[95.5 - 97.9]	0.0
**'KL0515'**	**'KL0867'**	96.4	[94.5 - 97.7]	86.9	[84.3 - 89.1]	96.8	[95.4 - 97.8]	0.01
**'KL0475'**	**'KL0515'**	91.1	[88.0 - 93.5]	86.9	[84.4 - 89.2]	93.0	[90.6 - 94.7]	0.0
**'KL0515'**	**'KL0796'**	94.2	[91.7 - 96.0]	86.9	[84.3 - 89.1]	95.2	[93.4 - 96.6]	0.03
**'KL0556'**	**'KL0785'**	96.8	[95.1 - 98.0]	86.9	[84.3 - 89.1]	97.1	[95.8 - 98.1]	0.04
**'KL0818'**	**'KL0825'**	96.1	[94.1 - 97.5]	86.9	[84.3 - 89.1]	96.6	[95.1 - 97.7]	0.05
**'KL0785'**	**'KL0825'**	96.0	[94.0 - 97.4]	86.9	[84.3 - 89.1]	96.6	[95.0 - 97.6]	0.05
**'KL0556'**	**'KL0867'**	97.5	[95.9 - 98.4]	86.9	[84.3 - 89.1]	97.6	[96.4 - 98.4]	0.02
**'KL0515'**	**'KL0547'**	95.5	[93.3 - 97.0]	86.9	[84.3 - 89.1]	96.2	[94.5 - 97.3]	0.02
**'KL0515'**	**'KL0540'**	97.5	[96.0 - 98.5]	86.9	[84.3 - 89.1]	97.6	[96.4 - 98.4]	0.03
**'KL0497'**	**'KL0556'**	98.3	[97.1 - 99.0]	86.9	[84.3 - 89.1]	98.2	[97.2 - 98.8]	0.02
**'KL0515'**	**'KL0556'**	97.9	[96.5 - 98.7]	86.8	[84.2 - 89.0]	97.9	[96.8 - 98.6]	0.0
**'KL0483'**	**'KL0853'**	97.1	[95.5 - 98.2]	86.8	[84.2 - 89.1]	97.4	[96.1 - 98.2]	0.01
**'KL0556'**	**'KL0846'**	98.7	[97.7 - 99.3]	86.8	[84.2 - 89.0]	98.5	[97.6 - 99.1]	0.01
**'KL0540'**	**'KL0870'**	95.9	[93.9 - 97.3]	86.8	[84.2 - 89.0]	96.5	[94.9 - 97.6]	0.01

Query	Subject	d_0	C.I. d_0	d_4	C.I. d_4	d_6	C.I. d_6	Diff. G+C Percent
**'KL0547'**	**'KL0818'**	94.5	[92.1 - 96.2]	86.8	[84.2 - 89.1]	95.5	[93.6 - 96.8]	0.01
**'KL0547'**	**'KL0556'**	96.6	[94.8 - 97.8]	86.8	[84.2 - 89.0]	97.0	[95.6 - 98.0]	0.02
**'KL0547'**	**'KL0785'**	94.4	[91.9 - 96.1]	86.8	[84.2 - 89.0]	95.4	[93.5 - 96.7]	0.02
**'KL0540'**	**'KL0556'**	98.5	[97.4 - 99.1]	86.8	[84.2 - 89.0]	98.4	[97.4 - 99.0]	0.04
**'KL0547'**	**'KL0853'**	94.7	[92.4 - 96.4]	86.8	[84.2 - 89.1]	95.6	[93.8 - 96.9]	0.07
**'KL0556'**	**'KL0818'**	96.9	[95.2 - 98.0]	86.8	[84.2 - 89.1]	97.2	[95.9 - 98.1]	0.04
**'KL0556'**	**'KL0853'**	95.8	[93.8 - 97.2]	86.8	[84.2 - 89.0]	96.4	[94.8 - 97.5]	0.09
**'KL0556'**	**'KL0796'**	95.3	[93.0 - 96.8]	86.7	[84.1 - 89.0]	96.0	[94.3 - 97.2]	0.03
**'KL0483'**	**'KL0556'**	96.0	[94.0 - 97.4]	86.7	[84.1 - 88.9]	96.5	[95.0 - 97.6]	0.08
**'KL0556'**	**'KL0840'**	98.8	[97.9 - 99.3]	86.7	[84.1 - 89.0]	98.6	[97.7 - 99.1]	0.01
**'KL0785'**	**'KL0867'**	95.5	[93.3 - 97.0]	86.7	[84.1 - 88.9]	96.2	[94.5 - 97.3]	0.03
**'KL0818'**	**'KL0867'**	95.6	[93.5 - 97.1]	86.7	[84.1 - 88.9]	96.2	[94.6 - 97.4]	0.02
**'KL0556'**	**'KL0825'**	98.0	[96.6 - 98.8]	86.6	[84.0 - 88.8]	97.9	[96.8 - 98.7]	0.01
**'KL0556'**	**'KL0832'**	98.9	[98.0 - 99.4]	86.6	[84.0 - 88.9]	98.6	[97.8 - 99.2]	0.01
**'KL0501'**	**'KL0515'**	98.2	[97.0 - 98.9]	86.5	[83.9 - 88.8]	98.1	[97.1 - 98.8]	0.02
**'KL0501'**	**'KL0818'**	97.3	[95.7 - 98.3]	86.5	[83.9 - 88.8]	97.5	[96.2 - 98.3]	0.05
**'KL0501'**	**'KL0785'**	97.2	[95.6 - 98.2]	86.5	[83.9 - 88.7]	97.4	[96.1 - 98.3]	0.06
**'KL0501'**	**'KL0556'**	98.9	[98.0 - 99.4]	86.5	[83.9 - 88.8]	98.6	[97.8 - 99.2]	0.01
**'KL0785'**	**'KL0796'**	95.6	[93.5 - 97.1]	86.4	[83.7 - 88.6]	96.2	[94.6 - 97.4]	0.01
**'KL0796'**	**'KL0818'**	95.7	[93.6 - 97.2]	86.4	[83.7 - 88.6]	96.3	[94.7 - 97.4]	0.01
**'KL0541'**	**'KL0818'**	94.6	[92.2 - 96.3]	86.3	[83.7 - 88.6]	95.4	[93.6 - 96.8]	0.01
**'KL0475'**	**'KL0785'**	92.8	[90.0 - 94.9]	86.3	[83.7 - 88.6]	94.1	[92.0 - 95.7]	0.04
**'KL0475'**	**'KL0818'**	93.0	[90.2 - 95.0]	86.3	[83.7 - 88.6]	94.2	[92.2 - 95.8]	0.04
**'KL0541'**	**'KL0785'**	94.4	[92.0 - 96.2]	86.3	[83.7 - 88.6]	95.4	[93.5 - 96.7]	0.0
**'KL0483'**	**'KL0818'**	96.6	[94.7 - 97.8]	86.3	[83.6 - 88.5]	96.9	[95.4 - 97.9]	0.04
**'KL0483'**	**'KL0785'**	96.4	[94.6 - 97.7]	86.2	[83.6 - 88.5]	96.8	[95.3 - 97.8]	0.03
**'KL0515'**	*Corynebacterium silvaticum* KL0182	91.6	[88.6 - 93.9]	41.1	[38.6 - 43.6]	81.5	[78.1 - 84.4]	1.12

Query	Subject	d_0	C.I. d_0	d_4	C.I. d_4	d_6	C.I. d_6	Diff. G+C Percent
**'KL0785'**	*Corynebacterium silvaticum* KL0182	90.3	[87.1 - 92.8]	41.1	[38.6 - 43.6]	80.3	[76.9 - 83.4]	1.08
**'KL0876'**	*Corynebacterium silvaticum* KL0182	90.9	[87.8 - 93.3]	41.1	[38.6 - 43.6]	80.9	[77.5 - 83.8]	1.17
**'KL0818'**	*Corynebacterium silvaticum* KL0182	90.3	[87.1 - 92.8]	41.1	[38.6 - 43.7]	80.4	[77.0 - 83.4]	1.09
**'KL0853'**	*Corynebacterium silvaticum* KL0182	87.8	[84.3 - 90.6]	41.1	[38.6 - 43.7]	78.2	[74.8 - 81.3]	1.04
**'KL0832'**	*Corynebacterium silvaticum* KL0182	91.6	[88.6 - 93.9]	40.9	[38.4 - 43.5]	81.4	[78.0 - 84.3]	1.14
**'KL0547'**	*Corynebacterium silvaticum* KL0182	88.9	[85.5 - 91.6]	40.9	[38.4 - 43.4]	79.0	[75.6 - 82.1]	1.11
**'KL0825'**	*Corynebacterium silvaticum* KL0182	90.1	[86.8 - 92.6]	40.9	[38.4 - 43.5]	80.1	[76.6 - 83.1]	1.14
**'KL0501'**	*Corynebacterium silvaticum* KL0182	91.5	[88.5 - 93.8]	40.9	[38.4 - 43.4]	81.3	[78.0 - 84.3]	1.14
**'KL0556'**	*Corynebacterium silvaticum* KL0182	92.7	[89.9 - 94.8]	40.9	[38.5 - 43.5]	82.4	[79.1 - 85.3]	1.13
**'KL0867'**	*Corynebacterium silvaticum* KL0182	90.0	[86.8 - 92.5]	40.9	[38.4 - 43.4]	80.0	[76.6 - 83.0]	1.11
**'KL0497'**	*Corynebacterium silvaticum* KL0182	91.4	[88.3 - 93.7]	40.9	[38.4 - 43.4]	81.2	[77.8 - 84.1]	1.14
**'KL0846'**	*Corynebacterium silvaticum* KL0182	91.5	[88.4 - 93.8]	40.9	[38.4 - 43.4]	81.2	[77.9 - 84.2]	1.13
**'KL0840'**	*Corynebacterium silvaticum* KL0182	91.6	[88.6 - 93.9]	40.9	[38.4 - 43.4]	81.4	[78.0 - 84.3]	1.14
**'KL0870'**	*Corynebacterium silvaticum* KL0182	88.5	[85.1 - 91.3]	40.9	[38.4 - 43.4]	78.7	[75.3 - 81.8]	1.08
**'KL0475'**	*Corynebacterium silvaticum* KL0182	83.5	[79.7 - 86.7]	40.8	[38.3 - 43.4]	74.6	[71.1 - 77.7]	1.13
**'KL0540'**	*Corynebacterium silvaticum* KL0182	91.6	[88.6 - 93.9]	40.7	[38.2 - 43.2]	81.3	[77.9 - 84.2]	1.09
**'KL0483'**	*Corynebacterium silvaticum* KL0182	88.2	[84.7 - 91.0]	40.7	[38.2 - 43.3]	78.4	[74.9 - 81.4]	1.05
**'KL0541'**	*Corynebacterium silvaticum* KL0182	86.4	[82.8 - 89.4]	40.7	[38.3 - 43.3]	76.9	[73.4 - 80.0]	1.08
**'KL0796'**	*Corynebacterium silvaticum* KL0182	87.8	[84.3 - 90.6]	40.7	[38.3 - 43.3]	78.1	[74.6 - 81.1]	1.1
**'KL0832'**	*Corynebacterium pseudotuberculosis* ATCC 19410	86.8	[83.2 - 89.7]	27.6	[25.2 - 30.1]	67.9	[64.5 - 71.1]	1.13
**'KL0515'**	*Corynebacterium pseudotuberculosis* ATCC 19410	86.7	[83.1 - 89.6]	27.6	[25.2 - 30.0]	67.8	[64.3 - 71.0]	1.14
**'KL0497'**	*Corynebacterium pseudotuberculosis* ATCC 19410	86.5	[82.8 - 89.4]	27.6	[25.2 - 30.0]	67.6	[64.2 - 70.8]	1.12
**'KL0840'**	*Corynebacterium pseudotuberculosis* DSM 20689	86.8	[83.2 - 89.7]	27.6	[25.2 - 30.1]	67.9	[64.4 - 71.1]	1.13
**'KL0483'**	*Corynebacterium pseudotuberculosis* ATCC 19410	82.2	[78.4 - 85.5]	27.6	[25.2 - 30.1]	64.6	[61.2 - 67.8]	1.21
**'KL0818'**	*Corynebacterium pseudotuberculosis* ATCC 19410	84.8	[81.1 - 87.9]	27.6	[25.2 - 30.1]	66.4	[63.0 - 69.6]	1.18

Query	Subject	d_0	C.I. d_0	d_4	C.I. d_4	d_6	C.I. d_6	Diff. G+C Percent
**'KL0541'**	*Corynebacterium pseudotuberculosis* ATCC 19410	80.5	[76.6 - 84.0]	27.6	[25.2 - 30.0]	63.4	[60.1 - 66.6]	1.18
**'KL0832'**	*Corynebacterium pseudotuberculosis* DSM 20689	86.8	[83.2 - 89.7]	27.6	[25.2 - 30.1]	67.9	[64.5 - 71.1]	1.12
**'KL0483'**	*Corynebacterium pseudotuberculosis* DSM 20689	82.2	[78.4 - 85.6]	27.6	[25.2 - 30.1]	64.6	[61.3 - 67.8]	1.21
**'KL0876'**	*Corynebacterium pseudotuberculosis* ATCC 19410	86.5	[82.8 - 89.4]	27.6	[25.3 - 30.1]	67.7	[64.2 - 70.9]	1.09
**'KL0853'**	*Corynebacterium pseudotuberculosis* ATCC 19410	83.5	[79.6 - 86.7]	27.6	[25.2 - 30.1]	65.5	[62.1 - 68.7]	1.23
**'KL0556'**	*Corynebacterium pseudotuberculosis* ATCC 19410	88.0	[84.6 - 90.8]	27.6	[25.2 - 30.0]	68.8	[65.3 - 72.0]	1.14
**'KL0497'**	*Corynebacterium pseudotuberculosis* DSM 20689	86.5	[82.9 - 89.4]	27.6	[25.2 - 30.1]	67.6	[64.2 - 70.9]	1.12
**'KL0853'**	*Corynebacterium pseudotuberculosis* DSM 20689	83.6	[79.8 - 86.8]	27.6	[25.2 - 30.1]	65.6	[62.2 - 68.8]	1.22
**'KL0846'**	*Corynebacterium pseudotuberculosis* DSM 20689	86.6	[83.0 - 89.6]	27.6	[25.2 - 30.1]	67.7	[64.3 - 71.0]	1.13
**'KL0515'**	*Corynebacterium pseudotuberculosis* DSM 20689	86.7	[83.1 - 89.6]	27.6	[25.2 - 30.0]	67.8	[64.4 - 71.0]	1.14
**'KL0796'**	*Corynebacterium pseudotuberculosis* ATCC 19410	81.4	[77.5 - 84.7]	27.6	[25.2 - 30.1]	64.0	[60.7 - 67.2]	1.17
**'KL0796'**	*Corynebacterium pseudotuberculosis* DSM 20689	81.4	[77.5 - 84.7]	27.6	[25.2 - 30.1]	64.0	[60.7 - 67.2]	1.17
**'KL0870'**	*Corynebacterium pseudotuberculosis* DSM 20689	82.6	[78.8 - 85.9]	27.6	[25.3 - 30.1]	64.9	[61.5 - 68.1]	1.18
**'KL0556'**	*Corynebacterium pseudotuberculosis* DSM 20689	88.0	[84.6 - 90.8]	27.6	[25.2 - 30.1]	68.8	[65.4 - 72.0]	1.14
**'KL0846'**	*Corynebacterium pseudotuberculosis* ATCC 19410	86.6	[83.0 - 89.6]	27.6	[25.2 - 30.1]	67.7	[64.3 - 71.0]	1.13
**'KL0840'**	*Corynebacterium pseudotuberculosis* ATCC 19410	86.8	[83.2 - 89.7]	27.6	[25.2 - 30.1]	67.8	[64.4 - 71.1]	1.13
**'KL0825'**	*Corynebacterium pseudotuberculosis* DSM 20689	85.1	[81.4 - 88.2]	27.6	[25.2 - 30.1]	66.7	[63.3 - 69.9]	1.13
**'KL0876'**	*Corynebacterium pseudotuberculosis* DSM 20689	86.5	[82.9 - 89.4]	27.6	[25.3 - 30.1]	67.7	[64.3 - 70.9]	1.09
**'KL0825'**	*Corynebacterium pseudotuberculosis* ATCC 19410	85.1	[81.4 - 88.2]	27.6	[25.2 - 30.1]	66.7	[63.3 - 69.9]	1.13
**'KL0541'**	*Corynebacterium pseudotuberculosis* DSM 20689	80.6	[76.6 - 84.0]	27.6	[25.2 - 30.0]	63.4	[60.1 - 66.6]	1.18
**'KL0870'**	*Corynebacterium pseudotuberculosis* ATCC 19410	82.6	[78.8 - 85.9]	27.6	[25.2 - 30.1]	64.9	[61.5 - 68.1]	1.19

Query	Subject	d_0	C.I. d_0	d_4	C.I. d_4	d_6	C.I. d_6	Diff. G+C Percent
**'KL0818'**	*Corynebacterium pseudotuberculosis* DSM 20689	84.9	[81.2 - 88.0]	27.6	[25.2 - 30.1]	66.5	[63.1 - 69.7]	1.17
**'KL0867'**	*Corynebacterium pseudotuberculosis* DSM 20689	85.1	[81.3 - 88.2]	27.5	[25.2 - 30.0]	66.6	[63.2 - 69.8]	1.15
**'KL0547'**	*Corynebacterium pseudotuberculosis* DSM 20689	83.7	[79.9 - 86.9]	27.5	[25.2 - 30.0]	65.6	[62.2 - 68.8]	1.16
**'KL0867'**	*Corynebacterium pseudotuberculosis* ATCC 19410	85.0	[81.3 - 88.1]	27.5	[25.2 - 30.0]	66.5	[63.2 - 69.8]	1.16
**'KL0785'**	*Corynebacterium pseudotuberculosis* ATCC 19410	85.3	[81.6 - 88.4]	27.5	[25.2 - 30.0]	66.7	[63.3 - 70.0]	1.18
**'KL0540'**	*Corynebacterium pseudotuberculosis* ATCC 19410	86.6	[83.0 - 89.6]	27.5	[25.2 - 30.0]	67.7	[64.3 - 70.9]	1.18
**'KL0785'**	*Corynebacterium pseudotuberculosis* DSM 20689	85.4	[81.7 - 88.5]	27.5	[25.2 - 30.0]	66.8	[63.4 - 70.1]	1.18
**'KL0501'**	*Corynebacterium pseudotuberculosis* ATCC 19410	86.9	[83.3 - 89.8]	27.5	[25.2 - 30.0]	67.9	[64.5 - 71.1]	1.13
**'KL0547'**	*Corynebacterium pseudotuberculosis* ATCC 19410	83.7	[79.9 - 86.9]	27.5	[25.2 - 30.0]	65.6	[62.2 - 68.8]	1.16
**'KL0501'**	*Corynebacterium pseudotuberculosis* DSM 20689	86.9	[83.3 - 89.8]	27.5	[25.2 - 30.0]	67.9	[64.5 - 71.2]	1.12
**'KL0540'**	*Corynebacterium pseudotuberculosis* DSM 20689	86.6	[83.0 - 89.6]	27.5	[25.2 - 30.0]	67.7	[64.3 - 71.0]	1.17
**'KL0475'**	*Corynebacterium pseudotuberculosis* DSM 20689	77.8	[73.8 - 81.3]	27.4	[25.1 - 29.9]	61.4	[58.2 - 64.6]	1.14
**'KL0475'**	*Corynebacterium pseudotuberculosis* ATCC 19410	77.8	[73.8 - 81.3]	27.4	[25.1 - 29.9]	61.4	[58.1 - 64.6]	1.14
**'KL0870'**	*Corynebacterium mustelae* DSM 45274	12.9	[10.2 - 16.2]	25.2	[22.9 - 27.7]	13.3	[11.0 - 16.1]	0.8
**'KL0876'**	*Corynebacterium mustelae* DSM 45274	13.0	[10.2 - 16.2]	25.2	[22.8 - 27.7]	13.3	[11.0 - 16.1]	0.71
**'KL0846'**	*Corynebacterium mustelae* DSM 45274	13.0	[10.2 - 16.2]	25.2	[22.8 - 27.6]	13.3	[11.0 - 16.1]	0.74
**'KL0867'**	*Corynebacterium mustelae* DSM 45274	13.0	[10.2 - 16.2]	25.2	[22.8 - 27.6]	13.3	[11.0 - 16.1]	0.77
**'KL0540'**	*Corynebacterium mustelae* DSM 45274	13.0	[10.2 - 16.2]	25.2	[22.9 - 27.7]	13.3	[11.0 - 16.1]	0.79
**'KL0541'**	*Corynebacterium mustelae* DSM 45274	12.9	[10.2 - 16.2]	25.2	[22.8 - 27.6]	13.3	[11.0 - 16.1]	0.79
**'KL0547'**	*Corynebacterium mustelae* DSM 45274	12.9	[10.2 - 16.2]	25.2	[22.8 - 27.6]	13.3	[11.0 - 16.1]	0.77
**'KL0853'**	*Corynebacterium mustelae* DSM 45274	12.9	[10.2 - 16.2]	25.2	[22.9 - 27.7]	13.3	[11.0 - 16.1]	0.84
**'KL0832'**	*Corynebacterium mustelae* DSM 45274	13.0	[10.2 - 16.2]	25.2	[22.9 - 27.7]	13.3	[11.0 - 16.1]	0.74
**'KL0556'**	*Corynebacterium mustelae* DSM 45274	13.0	[10.3 - 16.2]	25.2	[22.9 - 27.7]	13.3	[11.0 - 16.1]	0.75

Query	Subject	d_0	C.I. d_0	d_4	C.I. d_4	d_6	C.I. d_6	Diff. G+C Percent
**'KL0501'**	*Corynebacterium mustelae* DSM 45274	13.0	[10.2 - 16.2]	25.2	[22.9 - 27.7]	13.3	[11.0 - 16.1]	0.74
**'KL0483'**	*Corynebacterium mustelae* DSM 45274	12.9	[10.2 - 16.2]	25.2	[22.9 - 27.7]	13.3	[11.0 - 16.1]	0.83
**'KL0825'**	*Corynebacterium mustelae* DSM 45274	13.0	[10.2 - 16.2]	25.2	[22.9 - 27.7]	13.3	[11.0 - 16.1]	0.74
**'KL0840'**	*Corynebacterium mustelae* DSM 45274	13.0	[10.2 - 16.2]	25.2	[22.9 - 27.7]	13.3	[11.0 - 16.1]	0.74
**'KL0497'**	*Corynebacterium mustelae* DSM 45274	13.0	[10.2 - 16.2]	25.1	[22.8 - 27.6]	13.3	[11.0 - 16.1]	0.73
**'KL0796'**	*Corynebacterium mustelae* DSM 45274	12.9	[10.2 - 16.2]	25.1	[22.8 - 27.6]	13.3	[11.0 - 16.1]	0.78
**'KL0853'**	*Corynebacterium vitaeruminis* DSM 20294	13.2	[10.5 - 16.5]	25.0	[22.7 - 27.5]	13.6	[11.2 - 16.3]	12.12
**'KL0483'**	*Corynebacterium vitaeruminis* DSM 20294	13.2	[10.5 - 16.5]	25.0	[22.6 - 27.4]	13.6	[11.2 - 16.4]	12.13
**'KL0876'**	*Corynebacterium vitaeruminis* DSM 20294	13.2	[10.5 - 16.5]	25.0	[22.7 - 27.5]	13.6	[11.2 - 16.3]	12.25
**'KL0825'**	*Corynebacterium vitaeruminis* DSM 20294	13.2	[10.5 - 16.5]	24.9	[22.6 - 27.4]	13.6	[11.2 - 16.4]	12.21
**'KL0541'**	*Corynebacterium vitaeruminis* DSM 20294	13.2	[10.5 - 16.5]	24.9	[22.6 - 27.4]	13.6	[11.2 - 16.4]	12.16
**'KL0846'**	*Corynebacterium vitaeruminis* DSM 20294	13.2	[10.5 - 16.5]	24.9	[22.6 - 27.4]	13.6	[11.2 - 16.4]	12.21
**'KL0840'**	*Corynebacterium vitaeruminis* DSM 20294	13.2	[10.5 - 16.5]	24.9	[22.6 - 27.4]	13.6	[11.2 - 16.4]	12.21
**'KL0832'**	*Corynebacterium vitaeruminis* DSM 20294	13.2	[10.5 - 16.5]	24.9	[22.6 - 27.4]	13.6	[11.2 - 16.4]	12.22
**'KL0870'**	*Corynebacterium pseudopelargi* CCM 8832	13.2	[10.5 - 16.5]	24.9	[22.6 - 27.4]	13.6	[11.2 - 16.4]	4.54
**'KL0867'**	*Corynebacterium vitaeruminis* DSM 20294	13.2	[10.5 - 16.5]	24.8	[22.5 - 27.3]	13.6	[11.2 - 16.4]	12.19
**'KL0501'**	*Corynebacterium vitaeruminis* DSM 20294	13.2	[10.5 - 16.5]	24.8	[22.5 - 27.3]	13.6	[11.2 - 16.4]	12.22
**'KL0547'**	*Corynebacterium vitaeruminis* DSM 20294	13.2	[10.5 - 16.5]	24.8	[22.5 - 27.3]	13.6	[11.2 - 16.4]	12.18
**'KL0853'**	*Corynebacterium pelargi* DSM 46737	13.2	[10.5 - 16.5]	24.7	[22.4 - 27.1]	13.6	[11.2 - 16.3]	4.76
**'KL0876'**	*Corynebacterium pseudopelargi* CCM 8832	13.2	[10.5 - 16.5]	24.7	[22.4 - 27.2]	13.6	[11.2 - 16.4]	4.63
**'KL0515'**	*Corynebacterium vitaeruminis* DSM 20294	13.2	[10.5 - 16.5]	24.7	[22.3 - 27.1]	13.6	[11.2 - 16.4]	12.2
**'KL0870'**	*Corynebacterium vitaeruminis* DSM 20294	13.2	[10.5 - 16.5]	24.7	[22.4 - 27.1]	13.6	[11.2 - 16.4]	12.16
**'KL0853'**	*Corynebacterium pseudopelargi* CCM 8832	13.2	[10.5 - 16.5]	24.6	[22.3 - 27.1]	13.6	[11.2 - 16.4]	4.5
**'KL0556'**	*Corynebacterium vitaeruminis* DSM 20294	13.2	[10.5 - 16.5]	24.6	[22.3 - 27.1]	13.6	[11.2 - 16.4]	12.2
**'KL0540'**	*Corynebacterium vitaeruminis* DSM 20294	13.2	[10.5 - 16.5]	24.6	[22.3 - 27.0]	13.6	[11.2 - 16.4]	12.17
**'KL0796'**	*Corynebacterium vitaeruminis* DSM 20294	13.2	[10.5 - 16.5]	24.6	[22.3 - 27.1]	13.6	[11.2 - 16.4]	12.17
**'KL0840'**	*Corynebacterium pseudopelargi* CCM 8832	13.3	[10.5 - 16.6]	24.5	[22.1 - 26.9]	13.6	[11.2 - 16.4]	4.59

Query	Subject	d_0	C.I. d_0	d_4	C.I. d_4	d_6	C.I. d_6	Diff. G+C Percent
**'KL0547'**	*Corynebacterium pseudopelargi* CCM 8832	13.2	[10.5 - 16.6]	24.5	[22.2 - 26.9]	13.6	[11.2 - 16.4]	4.56
**'KL0825'**	*Corynebacterium pseudopelargi* CCM 8832	13.2	[10.5 - 16.6]	24.5	[22.1 - 26.9]	13.6	[11.2 - 16.4]	4.59
**'KL0501'**	*Corynebacterium pseudopelargi* CCM 8832	13.3	[10.5 - 16.6]	24.5	[22.2 - 27.0]	13.6	[11.3 - 16.4]	4.6
**'KL0832'**	*Corynebacterium pseudopelargi* CCM 8832	13.3	[10.5 - 16.6]	24.5	[22.1 - 26.9]	13.6	[11.2 - 16.4]	4.6
**'KL0501'**	*Corynebacterium pelargi* DSM 46737	13.3	[10.5 - 16.6]	24.5	[22.2 - 27.0]	13.6	[11.3 - 16.4]	4.86
**'KL0483'**	*Corynebacterium pseudopelargi* CCM 8832	13.2	[10.5 - 16.5]	24.5	[22.2 - 27.0]	13.6	[11.2 - 16.4]	4.51
**'KL0870'**	*Corynebacterium pelargi* DSM 46737	13.2	[10.5 - 16.5]	24.5	[22.2 - 26.9]	13.6	[11.2 - 16.4]	4.8
**'KL0497'**	*Corynebacterium pelargi* DSM 46737	13.3	[10.5 - 16.6]	24.5	[22.2 - 27.0]	13.6	[11.2 - 16.4]	4.87
**'KL0876'**	*Corynebacterium pelargi* DSM 46737	13.2	[10.5 - 16.5]	24.5	[22.1 - 26.9]	13.6	[11.2 - 16.4]	4.9
**'KL0497'**	*Corynebacterium vitaeruminis* DSM 20294	13.2	[10.5 - 16.6]	24.5	[22.2 - 27.0]	13.6	[11.2 - 16.4]	12.22
**'KL0541'**	*Corynebacterium pseudopelargi* CCM 8832	13.2	[10.5 - 16.5]	24.5	[22.1 - 26.9]	13.6	[11.2 - 16.4]	4.54
**'KL0540'**	*Corynebacterium pseudopelargi* CCM 8832	13.3	[10.5 - 16.6]	24.5	[22.2 - 26.9]	13.6	[11.2 - 16.4]	4.55
**'KL0867'**	*Corynebacterium pelargi* DSM 46737	13.3	[10.5 - 16.6]	24.4	[22.1 - 26.9]	13.6	[11.2 - 16.4]	4.83
**'KL0785'**	*Corynebacterium vitaeruminis* DSM 20294	13.2	[10.5 - 16.5]	24.4	[22.1 - 26.9]	13.6	[11.2 - 16.4]	12.16
**'KL0818'**	*Corynebacterium vitaeruminis* DSM 20294	13.2	[10.5 - 16.5]	24.4	[22.1 - 26.9]	13.6	[11.2 - 16.4]	12.17
**'KL0825'**	*Corynebacterium pelargi* DSM 46737	13.3	[10.5 - 16.6]	24.4	[22.1 - 26.9]	13.6	[11.2 - 16.4]	4.86
**'KL0840'**	*Corynebacterium pelargi* DSM 46737	13.3	[10.5 - 16.6]	24.4	[22.1 - 26.9]	13.6	[11.3 - 16.4]	4.86
**'KL0475'**	*Corynebacterium mustelae* DSM 45274	12.8	[10.1 - 16.1]	24.4	[22.1 - 26.9]	13.2	[10.8 - 15.9]	0.75
**'KL0540'**	*Corynebacterium pelargi* DSM 46737	13.3	[10.5 - 16.6]	24.4	[22.1 - 26.9]	13.6	[11.3 - 16.4]	4.81
**'KL0846'**	*Corynebacterium pelargi* DSM 46737	13.3	[10.5 - 16.6]	24.4	[22.1 - 26.9]	13.6	[11.3 - 16.4]	4.86
**'KL0483'**	*Corynebacterium pelargi* DSM 46737	13.3	[10.5 - 16.6]	24.4	[22.1 - 26.9]	13.6	[11.2 - 16.4]	4.78
**'KL0796'**	*Corynebacterium pelargi* DSM 46737	13.2	[10.5 - 16.6]	24.4	[22.1 - 26.9]	13.6	[11.2 - 16.4]	4.82
**'KL0541'**	*Corynebacterium pelargi* DSM 46737	13.2	[10.5 - 16.6]	24.4	[22.1 - 26.9]	13.6	[11.2 - 16.4]	4.81
**'KL0556'**	*Corynebacterium kutscheri* DSM 20755	13.1	[10.4 - 16.4]	24.4	[22.1 - 26.8]	13.5	[11.1 - 16.3]	6.86
**'KL0832'**	*Corynebacterium pelargi* DSM 46737	13.3	[10.5 - 16.6]	24.4	[22.1 - 26.9]	13.6	[11.3 - 16.4]	4.86
**'KL0846'**	*Corynebacterium pseudopelargi* CCM 8832	13.3	[10.5 - 16.6]	24.4	[22.1 - 26.9]	13.6	[11.2 - 16.4]	4.59
**'KL0547'**	*Corynebacterium pelargi* DSM 46737	13.3	[10.5 - 16.6]	24.4	[22.1 - 26.9]	13.6	[11.2 - 16.4]	4.83

Query	Subject	d_0	C.I. d_0	d_4	C.I. d_4	d_6	C.I. d_6	Diff. G+C Percent
**'KL0796'**	*Corynebacterium pseudopelargi* CCM 8832	13.2	[10.5 - 16.6]	24.3	[21.9 - 26.7]	13.6	[11.2 - 16.4]	4.55
**'KL0497'**	*Corynebacterium pseudopelargi* CCM 8832	13.3	[10.5 - 16.6]	24.3	[22.0 - 26.8]	13.6	[11.3 - 16.4]	4.6
**'KL0867'**	*Corynebacterium pseudopelargi* CCM 8832	13.3	[10.5 - 16.6]	24.3	[22.0 - 26.7]	13.6	[11.2 - 16.4]	4.57
**'KL0515'**	*Corynebacterium mustelae* DSM 45274	13.0	[10.3 - 16.3]	24.1	[21.8 - 26.6]	13.4	[11.0 - 16.1]	0.75
**'KL0818'**	*Corynebacterium mustelae* DSM 45274	13.0	[10.3 - 16.3]	24.1	[21.8 - 26.5]	13.4	[11.0 - 16.1]	0.79
**'KL0785'**	*Corynebacterium mustelae* DSM 45274	13.0	[10.3 - 16.3]	24.1	[21.8 - 26.5]	13.4	[11.0 - 16.1]	0.79
**'KL0556'**	*Corynebacterium pseudopelargi* CCM 8832	13.2	[10.5 - 16.6]	24.1	[21.8 - 26.6]	13.6	[11.2 - 16.4]	4.59
**'KL0515'**	*Corynebacterium pseudopelargi* CCM 8832	13.3	[10.5 - 16.6]	24.0	[21.7 - 26.5]	13.6	[11.2 - 16.4]	4.58
**'KL0556'**	*Corynebacterium pelargi* DSM 46737	13.3	[10.5 - 16.6]	24.0	[21.7 - 26.5]	13.6	[11.3 - 16.4]	4.85
**'KL0818'**	*Corynebacterium pseudopelargi* CCM 8832	13.2	[10.5 - 16.6]	24.0	[21.7 - 26.4]	13.6	[11.2 - 16.4]	4.55
**'KL0785'**	*Corynebacterium pseudopelargi* CCM 8832	13.2	[10.5 - 16.6]	24.0	[21.7 - 26.5]	13.6	[11.2 - 16.4]	4.54
**'KL0853'**	*Corynebacterium kutscheri* DSM 20755	13.2	[10.5 - 16.5]	24.0	[21.7 - 26.5]	13.6	[11.2 - 16.3]	6.94
**'KL0825'**	*Corynebacterium kutscheri* DSM 20755	13.2	[10.5 - 16.5]	23.9	[21.6 - 26.4]	13.5	[11.2 - 16.3]	6.85
**'KL0483'**	*Corynebacterium kutscheri* DSM 20755	13.2	[10.4 - 16.5]	23.9	[21.6 - 26.4]	13.5	[11.2 - 16.3]	6.93
**'KL0832'**	*Corynebacterium kutscheri* DSM 20755	13.2	[10.5 - 16.5]	23.9	[21.6 - 26.4]	13.6	[11.2 - 16.3]	6.85
**'KL0867'**	*Corynebacterium kutscheri* DSM 20755	13.2	[10.5 - 16.5]	23.9	[21.6 - 26.4]	13.5	[11.2 - 16.3]	6.88
**'KL0497'**	*Corynebacterium kutscheri* DSM 20755	13.2	[10.5 - 16.5]	23.9	[21.6 - 26.4]	13.6	[11.2 - 16.3]	6.84
**'KL0501'**	*Corynebacterium kutscheri* DSM 20755	13.2	[10.5 - 16.5]	23.9	[21.6 - 26.4]	13.6	[11.2 - 16.3]	6.84
**'KL0540'**	*Corynebacterium kutscheri* DSM 20755	13.2	[10.5 - 16.5]	23.9	[21.6 - 26.4]	13.6	[11.2 - 16.3]	6.9
**'KL0840'**	*Corynebacterium kutscheri* DSM 20755	13.2	[10.5 - 16.5]	23.9	[21.6 - 26.4]	13.6	[11.2 - 16.3]	6.85
**'KL0796'**	*Corynebacterium kutscheri* DSM 20755	13.2	[10.4 - 16.5]	23.9	[21.6 - 26.4]	13.5	[11.2 - 16.3]	6.89
**'KL0846'**	*Corynebacterium kutscheri* DSM 20755	13.2	[10.5 - 16.5]	23.9	[21.6 - 26.4]	13.6	[11.2 - 16.3]	6.85
**'KL0870'**	*Corynebacterium kutscheri* DSM 20755	13.2	[10.5 - 16.5]	23.8	[21.5 - 26.2]	13.6	[11.2 - 16.3]	6.9
**'KL0547'**	*Corynebacterium kutscheri* DSM 20755	13.2	[10.5 - 16.5]	23.7	[21.4 - 26.2]	13.6	[11.2 - 16.4]	6.88
**'KL0475'**	*Corynebacterium phocae* DSM 44612	12.7	[10.1 - 16.0]	23.7	[21.4 - 26.2]	13.1	[10.8 - 15.9]	5.49
**'KL0541'**	*Corynebacterium kutscheri* DSM 20755	13.2	[10.5 - 16.5]	23.7	[21.4 - 26.2]	13.6	[11.2 - 16.4]	6.9
**'KL0515'**	*Corynebacterium kutscheri* DSM 20755	13.2	[10.5 - 16.5]	23.6	[21.3 - 26.1]	13.6	[11.2 - 16.4]	6.86

Query	Subject	d_0	C.I. d_0	d_4	C.I. d_4	d_6	C.I. d_6	Diff. G+C Percent
**'KL0785'**	*Corynebacterium kutscheri* DSM 20755	13.2	[10.5 - 16.5]	23.6	[21.3 - 26.1]	13.6	[11.2 - 16.3]	6.9
**'KL0876'**	*Corynebacterium kutscheri* DSM 20755	13.2	[10.5 - 16.5]	23.6	[21.3 - 26.0]	13.6	[11.2 - 16.4]	6.81
**'KL0818'**	*Corynebacterium kutscheri* DSM 20755	13.2	[10.5 - 16.5]	23.6	[21.3 - 26.1]	13.6	[11.2 - 16.3]	6.89
**'KL0556'**	*Corynebacterium phocae* DSM 44612	12.9	[10.2 - 16.2]	23.5	[21.2 - 25.9]	13.3	[10.9 - 16.0]	5.49
**'KL0475'**	*Corynebacterium vitaeruminis* DSM 20294	13.0	[10.3 - 16.3]	23.4	[21.1 - 25.8]	13.4	[11.0 - 16.2]	12.2
**'KL0475'**	*Corynebacterium pelargi* DSM 46737	13.0	[10.3 - 16.3]	23.4	[21.1 - 25.8]	13.4	[11.0 - 16.2]	4.85
**'KL0785'**	*Corynebacterium pelargi* DSM 46737	13.3	[10.6 - 16.6]	23.3	[21.0 - 25.7]	13.6	[11.3 - 16.4]	4.81
**'KL0515'**	*Corynebacterium pelargi* DSM 46737	13.3	[10.6 - 16.6]	23.3	[21.0 - 25.7]	13.7	[11.3 - 16.4]	4.85
**'KL0876'**	*Corynebacterium phocae* DSM 44612	12.9	[10.2 - 16.2]	23.3	[21.0 - 25.8]	13.3	[10.9 - 16.0]	5.53
**'KL0818'**	*Corynebacterium pelargi* DSM 46737	13.3	[10.6 - 16.6]	23.3	[21.0 - 25.7]	13.6	[11.3 - 16.4]	4.82
**'KL0475'**	*Corynebacterium kutscheri* DSM 20755	12.9	[10.2 - 16.2]	23.3	[21.0 - 25.8]	13.3	[11.0 - 16.1]	6.86
**'KL0853'**	*Corynebacterium phocae* DSM 44612	12.9	[10.2 - 16.2]	23.2	[20.9 - 25.6]	13.3	[10.9 - 16.0]	5.4
**'KL0475'**	*Corynebacterium pseudopelargi* CCM 8832	13.0	[10.3 - 16.3]	23.2	[20.9 - 25.6]	13.4	[11.0 - 16.2]	4.59
**'KL0785'**	*Corynebacterium phocae* DSM 44612	12.9	[10.2 - 16.2]	23.2	[20.9 - 25.6]	13.3	[10.9 - 16.0]	5.45
**'KL0818'**	*Corynebacterium phocae* DSM 44612	12.9	[10.2 - 16.2]	23.2	[20.9 - 25.6]	13.3	[10.9 - 16.0]	5.45
**'KL0853'**	*Corynebacterium diphtheriae* NCTC 11397	14.2	[11.3 - 17.5]	23.2	[20.9 - 25.6]	14.4	[12.0 - 17.3]	0.12
**'KL0515'**	*Corynebacterium phocae* DSM 44612	12.9	[10.2 - 16.2]	23.2	[20.9 - 25.6]	13.3	[10.9 - 16.0]	5.49
**'KL0818'**	*Corynebacterium diphtheriae* NCTC 11397	14.2	[11.4 - 17.6]	23.1	[20.8 - 25.6]	14.5	[12.0 - 17.3]	0.17
**'KL0515'**	*Corynebacterium diphtheriae* NCTC 11397	14.2	[11.4 - 17.6]	23.1	[20.8 - 25.6]	14.5	[12.0 - 17.3]	0.2
**'KL0785'**	*Corynebacterium diphtheriae* NCTC 11397	14.2	[11.4 - 17.6]	23.1	[20.8 - 25.6]	14.5	[12.0 - 17.3]	0.16
**'KL0547'**	*Corynebacterium phocae* DSM 44612	12.9	[10.2 - 16.2]	23.0	[20.7 - 25.5]	13.3	[10.9 - 16.0]	5.47
**'KL0540'**	*Corynebacterium diphtheriae* NCTC 11397	14.2	[11.4 - 17.6]	23.0	[20.7 - 25.4]	14.5	[12.0 - 17.3]	0.16
**'KL0825'**	*Corynebacterium diphtheriae* NCTC 11397	14.2	[11.4 - 17.6]	23.0	[20.7 - 25.4]	14.5	[12.0 - 17.3]	0.21
**'KL0846'**	*Corynebacterium diphtheriae* NCTC 11397	14.2	[11.4 - 17.6]	23.0	[20.7 - 25.4]	14.5	[12.0 - 17.3]	0.21
**'KL0541'**	*Corynebacterium phocae* DSM 44612	12.9	[10.2 - 16.2]	23.0	[20.7 - 25.5]	13.3	[10.9 - 16.0]	5.44
**'KL0832'**	*Corynebacterium diphtheriae* NCTC 11397	14.2	[11.4 - 17.6]	23.0	[20.7 - 25.4]	14.5	[12.0 - 17.3]	0.22
**'KL0515'**	*Corynebacterium diphtheriae* subsp. *lausannense* CHUV2995	14.1	[11.3 - 17.4]	23.0	[20.7 - 25.4]	14.4	[11.9 - 17.2]	0.62

Query	Subject	d_0	C.I. d_0	d_4	C.I. d_4	d_6	C.I. d_6	Diff. G+C Percent
**'KL0870'**	*Corynebacterium diphtheriae* NCTC 11397	14.2	[11.4 - 17.6]	23.0	[20.7 - 25.5]	14.5	[12.0 - 17.3]	0.16
**'KL0840'**	*Corynebacterium diphtheriae* NCTC 11397	14.2	[11.4 - 17.6]	23.0	[20.7 - 25.4]	14.5	[12.0 - 17.3]	0.21
**'KL0483'**	*Corynebacterium diphtheriae* NCTC 11397	14.2	[11.3 - 17.5]	23.0	[20.7 - 25.4]	14.4	[12.0 - 17.3]	0.13
**'KL0556'**	*Corynebacterium diphtheriae* NCTC 11397	14.2	[11.4 - 17.6]	23.0	[20.7 - 25.4]	14.5	[12.0 - 17.3]	0.2
**'KL0867'**	*Corynebacterium phocae* DSM 44612	12.9	[10.2 - 16.2]	23.0	[20.7 - 25.5]	13.3	[10.9 - 16.0]	5.47
**'KL0796'**	*Corynebacterium diphtheriae* NCTC 11397	14.2	[11.4 - 17.5]	22.9	[20.7 - 25.4]	14.4	[12.0 - 17.3]	0.17
**'KL0497'**	*Corynebacterium diphtheriae* NCTC 11397	14.2	[11.4 - 17.6]	22.9	[20.6 - 25.4]	14.5	[12.0 - 17.3]	0.22
**'KL0501'**	*Corynebacterium diphtheriae* NCTC 11397	14.2	[11.4 - 17.6]	22.9	[20.6 - 25.4]	14.5	[12.0 - 17.3]	0.22
**'KL0870'**	*Corynebacterium diphtheriae* subsp. *lausannense* CHUV2995	14.3	[11.5 - 17.7]	22.9	[20.6 - 25.4]	14.6	[12.1 - 17.4]	0.57
**'KL0501'**	*Corynebacterium phocae* DSM 44612	12.9	[10.2 - 16.2]	22.9	[20.7 - 25.4]	13.3	[10.9 - 16.0]	5.5
**'KL0785'**	*Corynebacterium diphtheriae* subsp. *lausannense* CHUV2995	14.1	[11.3 - 17.4]	22.9	[20.6 - 25.4]	14.4	[11.9 - 17.2]	0.58
**'KL0870'**	*Corynebacterium phocae* DSM 44612	12.9	[10.2 - 16.2]	22.9	[20.7 - 25.4]	13.3	[10.9 - 16.0]	5.44
**'KL0846'**	*Corynebacterium phocae* DSM 44612	12.9	[10.2 - 16.2]	22.9	[20.6 - 25.4]	13.3	[10.9 - 16.0]	5.5
**'KL0870'**	*Corynebacterium rouxii* FRC0190 T	14.5	[11.7 - 17.9]	22.9	[20.6 - 25.4]	14.7	[12.3 - 17.6]	0.14
**'KL0818'**	*Corynebacterium diphtheriae* subsp. *lausannense* CHUV2995	14.1	[11.3 - 17.4]	22.9	[20.6 - 25.4]	14.4	[11.9 - 17.2]	0.58
**'KL0547'**	*Corynebacterium diphtheriae* NCTC 11397	14.2	[11.4 - 17.6]	22.9	[20.6 - 25.4]	14.5	[12.0 - 17.3]	0.18
**'KL0876'**	*Corynebacterium diphtheriae* NCTC 11397	14.2	[11.4 - 17.6]	22.9	[20.7 - 25.4]	14.5	[12.1 - 17.4]	0.25
**'KL0497'**	*Corynebacterium phocae* DSM 44612	12.9	[10.2 - 16.2]	22.8	[20.5 - 25.3]	13.3	[10.9 - 16.0]	5.51
**'KL0540'**	*Corynebacterium rouxii* FRC0190 T	14.5	[11.6 - 17.9]	22.8	[20.5 - 25.2]	14.7	[12.3 - 17.6]	0.13
**'KL0541'**	*Corynebacterium diphtheriae* NCTC 11397	14.1	[11.3 - 17.5]	22.8	[20.5 - 25.3]	14.4	[12.0 - 17.3]	0.16
**'KL0867'**	*Corynebacterium diphtheriae* NCTC 11397	14.2	[11.4 - 17.6]	22.8	[20.5 - 25.3]	14.5	[12.0 - 17.3]	0.19
**'KL0483'**	*Corynebacterium rouxii* FRC0190 T	14.4	[11.6 - 17.8]	22.8	[20.5 - 25.2]	14.7	[12.2 - 17.5]	0.17
**'KL0796'**	*Corynebacterium rouxii* FRC0190 T	14.5	[11.6 - 17.9]	22.8	[20.6 - 25.3]	14.7	[12.3 - 17.6]	0.12
**'KL0483'**	*Corynebacterium phocae* DSM 44612	12.9	[10.2 - 16.2]	22.8	[20.5 - 25.2]	13.3	[10.9 - 16.0]	5.41
**'KL0540'**	*Corynebacterium phocae* DSM 44612	12.9	[10.2 - 16.2]	22.8	[20.6 - 25.3]	13.3	[10.9 - 16.0]	5.45
**'KL0497'**	*Corynebacterium rouxii* FRC0190 T	14.4	[11.6 - 17.8]	22.7	[20.5 - 25.2]	14.7	[12.2 - 17.5]	0.08

Query	Subject	d_0	C.I. d_0	d_4	C.I. d_4	d_6	C.I. d_6	Diff. G+C Percent
**'KL0832'**	*Corynebacterium phocae* DSM 44612	12.9	[10.2 - 16.2]	22.7	[20.4 - 25.2]	13.3	[10.9 - 16.1]	5.5
**'KL0556'**	*Corynebacterium rouxii* FRC0190 T	14.3	[11.5 - 17.7]	22.7	[20.4 - 25.1]	14.6	[12.1 - 17.4]	0.09
**'KL0796'**	*Corynebacterium phocae* DSM 44612	12.9	[10.2 - 16.2]	22.7	[20.5 - 25.2]	13.3	[10.9 - 16.0]	5.46
**'KL0541'**	*Corynebacterium rouxii* FRC0190 T	14.6	[11.7 - 18.0]	22.7	[20.4 - 25.1]	14.8	[12.3 - 17.7]	0.14
**'KL0515'**	*Corynebacterium rouxii* FRC0190 T	14.3	[11.5 - 17.7]	22.7	[20.4 - 25.1]	14.6	[12.1 - 17.4]	0.1
**'KL0825'**	*Corynebacterium phocae* DSM 44612	12.9	[10.2 - 16.2]	22.7	[20.5 - 25.2]	13.3	[10.9 - 16.0]	5.5
**'KL0785'**	*Corynebacterium rouxii* FRC0190 T	14.3	[11.5 - 17.7]	22.7	[20.5 - 25.2]	14.5	[12.1 - 17.4]	0.14
**'KL0818'**	*Corynebacterium rouxii* FRC0190 T	14.3	[11.5 - 17.7]	22.7	[20.4 - 25.2]	14.5	[12.1 - 17.4]	0.13
**'KL0840'**	*Corynebacterium phocae* DSM 44612	12.9	[10.2 - 16.2]	22.7	[20.4 - 25.2]	13.3	[10.9 - 16.1]	5.5
**'KL0547'**	*Corynebacterium rouxii* FRC0190 T	14.5	[11.7 - 17.9]	22.6	[20.3 - 25.1]	14.8	[12.3 - 17.6]	0.11
**'KL0853'**	*Corynebacterium diphtheriae* subsp. *lausannense* CHUV2995	14.1	[11.3 - 17.5]	22.6	[20.3 - 25.0]	14.4	[12.0 - 17.2]	0.53
**'KL0853'**	*Corynebacterium rouxii* FRC0190 T	14.4	[11.6 - 17.8]	22.6	[20.3 - 25.0]	14.7	[12.2 - 17.5]	0.18
**'KL0547'**	*Corynebacterium diphtheriae* subsp. *lausannense* CHUV2995	14.2	[11.3 - 17.5]	22.6	[20.3 - 25.0]	14.4	[12.0 - 17.3]	0.6
**'KL0796'**	*Corynebacterium diphtheriae* subsp. *lausannense* CHUV2995	14.2	[11.4 - 17.6]	22.6	[20.3 - 25.1]	14.5	[12.1 - 17.3]	0.59
**'KL0867'**	*Corynebacterium rouxii* FRC0190 T	14.4	[11.6 - 17.8]	22.6	[20.3 - 25.0]	14.7	[12.2 - 17.5]	0.11
**'KL0501'**	*Corynebacterium rouxii* FRC0190 T	14.4	[11.6 - 17.8]	22.5	[20.2 - 24.9]	14.6	[12.2 - 17.5]	0.08
**'KL0556'**	*Corynebacterium diphtheriae* subsp. *lausannense* CHUV2995	14.2	[11.3 - 17.5]	22.5	[20.2 - 24.9]	14.4	[12.0 - 17.3]	0.62
**'KL0846'**	*Corynebacterium rouxii* FRC0190 T	14.4	[11.6 - 17.8]	22.5	[20.3 - 25.0]	14.7	[12.2 - 17.5]	0.09
**'KL0497'**	*Corynebacterium diphtheriae* subsp. *lausannense* CHUV2995	14.2	[11.4 - 17.6]	22.5	[20.2 - 24.9]	14.5	[12.0 - 17.3]	0.64
**'KL0832'**	*Corynebacterium rouxii* FRC0190 T	14.4	[11.6 - 17.8]	22.5	[20.2 - 25.0]	14.7	[12.2 - 17.5]	0.08
**'KL0840'**	*Corynebacterium rouxii* FRC0190 T	14.4	[11.6 - 17.8]	22.5	[20.3 - 25.0]	14.7	[12.2 - 17.5]	0.08
**'KL0825'**	*Corynebacterium rouxii* FRC0190 T	14.4	[11.6 - 17.8]	22.5	[20.2 - 25.0]	14.6	[12.2 - 17.5]	0.08
**'KL0870'**	*Corynebacterium belfantii* FRC0043	14.3	[11.5 - 17.7]	22.5	[20.2 - 25.0]	14.6	[12.1 - 17.4]	0.26
**'KL0867'**	*Corynebacterium diphtheriae* subsp. *lausannense* CHUV2995	14.2	[11.3 - 17.5]	22.5	[20.2 - 24.9]	14.4	[12.0 - 17.3]	0.6
**'KL0501'**	*Corynebacterium diphtheriae* subsp. *lausannense* CHUV2995	14.2	[11.3 - 17.5]	22.4	[20.1 - 24.8]	14.4	[12.0 - 17.3]	0.63

Query	Subject	d_0	C.I. d_0	d_4	C.I. d_4	d_6	C.I. d_6	Diff. G+C Percent
**'KL0515'**	*Corynebacterium belfantii* FRC0043	14.1	[11.3 - 17.5]	22.4	[20.1 - 24.8]	14.4	[12.0 - 17.2]	0.3
**'KL0483'**	*Corynebacterium diphtheriae* subsp. *lausannense* CHUV2995	14.2	[11.4 - 17.6]	22.4	[20.1 - 24.8]	14.5	[12.0 - 17.3]	0.54
**'KL0840'**	*Corynebacterium diphtheriae* subsp. *lausannense* CHUV2995	14.1	[11.3 - 17.5]	22.4	[20.2 - 24.9]	14.4	[12.0 - 17.3]	0.63
**'KL0541'**	*Corynebacterium diphtheriae* subsp. *lausannense* CHUV2995	14.2	[11.4 - 17.6]	22.4	[20.1 - 24.9]	14.5	[12.0 - 17.3]	0.58
**'KL0818'**	*Corynebacterium belfantii* FRC0043	14.1	[11.3 - 17.5]	22.4	[20.1 - 24.8]	14.4	[12.0 - 17.2]	0.27
**'KL0825'**	*Corynebacterium diphtheriae* subsp. *lausannense* CHUV2995	14.1	[11.3 - 17.5]	22.4	[20.2 - 24.9]	14.4	[12.0 - 17.2]	0.63
**'KL0832'**	*Corynebacterium diphtheriae* subsp. *lausannense* CHUV2995	14.1	[11.3 - 17.5]	22.4	[20.2 - 24.9]	14.4	[12.0 - 17.3]	0.63
**'KL0846'**	*Corynebacterium diphtheriae* subsp. *lausannense* CHUV2995	14.2	[11.3 - 17.5]	22.4	[20.2 - 24.9]	14.4	[12.0 - 17.3]	0.63
**'KL0785'**	*Corynebacterium belfantii* FRC0043	14.1	[11.3 - 17.5]	22.4	[20.1 - 24.8]	14.4	[12.0 - 17.2]	0.26
**'KL0876'**	*Corynebacterium rouxii* FRC0190 T	14.4	[11.5 - 17.7]	22.4	[20.2 - 24.9]	14.6	[12.2 - 17.4]	0.05
**'KL0876'**	*Corynebacterium diphtheriae* subsp. *lausannense* CHUV2995	14.2	[11.4 - 17.5]	22.4	[20.1 - 24.9]	14.4	[12.0 - 17.3]	0.66
**'KL0540'**	*Corynebacterium diphtheriae* subsp. *lausannense* CHUV2995	14.2	[11.4 - 17.6]	22.3	[20.1 - 24.8]	14.5	[12.1 - 17.3]	0.58
**'KL0853'**	*Corynebacterium belfantii* FRC0043	14.2	[11.3 - 17.5]	22.2	[19.9 - 24.6]	14.4	[12.0 - 17.3]	0.22
**'KL0796'**	*Corynebacterium belfantii* FRC0043	14.3	[11.4 - 17.6]	22.1	[19.8 - 24.5]	14.5	[12.1 - 17.4]	0.27
**'KL0825'**	*Corynebacterium belfantii* FRC0043	14.2	[11.3 - 17.5]	22.0	[19.7 - 24.4]	14.4	[12.0 - 17.3]	0.31
**'KL0547'**	*Corynebacterium belfantii* FRC0043	14.2	[11.4 - 17.6]	22.0	[19.7 - 24.4]	14.5	[12.0 - 17.3]	0.28
**'KL0832'**	*Corynebacterium belfantii* FRC0043	14.2	[11.4 - 17.6]	22.0	[19.7 - 24.4]	14.4	[12.0 - 17.3]	0.32
**'KL0876'**	*Corynebacterium belfantii* FRC0043	14.2	[11.4 - 17.6]	22.0	[19.7 - 24.4]	14.5	[12.0 - 17.3]	0.35
**'KL0840'**	*Corynebacterium belfantii* FRC0043	14.2	[11.4 - 17.6]	22.0	[19.7 - 24.4]	14.4	[12.0 - 17.3]	0.31
**'KL0846'**	*Corynebacterium belfantii* FRC0043	14.2	[11.4 - 17.6]	22.0	[19.7 - 24.4]	14.4	[12.0 - 17.3]	0.31
**'KL0867'**	*Corynebacterium belfantii* FRC0043	14.2	[11.4 - 17.6]	21.9	[19.7 - 24.4]	14.5	[12.0 - 17.3]	0.29
**'KL0501'**	*Corynebacterium belfantii* FRC0043	14.2	[11.4 - 17.6]	21.9	[19.7 - 24.4]	14.4	[12.0 - 17.3]	0.32
**'KL0483'**	*Corynebacterium belfantii* FRC0043	14.2	[11.4 - 17.6]	21.9	[19.7 - 24.4]	14.5	[12.0 - 17.3]	0.23
**'KL0541'**	*Corynebacterium belfantii* FRC0043	14.2	[11.4 - 17.6]	21.9	[19.7 - 24.4]	14.5	[12.0 - 17.3]	0.26
**'KL0556'**	*Corynebacterium belfantii* FRC0043	14.2	[11.4 - 17.6]	21.9	[19.7 - 24.4]	14.5	[12.0 - 17.3]	0.3

Query	Subject	d_0	C.I. d_0	d_4	C.I. d_4	d_6	C.I. d_6	Diff. G+C Percent
**'KL0475'**	*Corynebacterium diphtheriae* NCTC 11397	13.9	[11.1 - 17.3]	21.9	[19.6 - 24.3]	14.2	[11.8 - 17.0]	0.2
**'KL0497'**	*Corynebacterium belfantii* FRC0043	14.2	[11.4 - 17.6]	21.9	[19.7 - 24.4]	14.5	[12.1 - 17.3]	0.32
**'KL0540'**	*Corynebacterium belfantii* FRC0043	14.3	[11.5 - 17.7]	21.8	[19.6 - 24.3]	14.5	[12.1 - 17.4]	0.27
**'KL0475'**	*Corynebacterium diphtheriae* subsp. *lausannense* CHUV2995	14.0	[11.2 - 17.3]	21.7	[19.4 - 24.1]	14.2	[11.8 - 17.1]	0.62
**'KL0475'**	*Corynebacterium rouxii* FRC0190 T	14.2	[11.4 - 17.6]	21.6	[19.3 - 24.0]	14.4	[12.0 - 17.3]	0.09
**'KL0475'**	*Corynebacterium belfantii* FRC0043	14.0	[11.2 - 17.3]	21.0	[18.7 - 23.4]	14.2	[11.8 - 17.1]	0.31

Table 4: Strains in your dataset

Joint dataset of automatically determined closest type strains (if this mode was chosen), manually selected type strains (if selected accordingly) and the provided user strains, if provided (marked in yellow).

Strain	Authority	Other deposits	Synonyms	Base pairs	Percent G+C	No. proteins	Goldstamp	Bioproject accession	Biosample accession	Assembly accession	IMG OID
Corynebacterium vitaeruminis DSM 20294	(Bechdel et al. 1928) Lanéelle et al. 1980	CCUG 28792; ATCC 10234; JCM 1323; IFO 12143; NBRC 12143; VKM B-1211; CIP 82.07; NCIB 9291; NCIMB 9291	*Brevibacterium vitaeruminis*; *Corynebacterium vitaeruminis*; *Flavobacterium vitarumen*	2931 780	65.5	2577	Gp0023683	PRJNA172966	SAMN03081455	GCA_000550805	2558860221
Corynebacterium diphtheriae subsp. *lausannense* CHUV2995	Tagini et al. 2019	CCUG 72509; DSM 107520	*Corynebacterium diphtheriae* subsp. *lausannense*	3060 363	53.9	3145	Gp0442955	PRJEB24256	SAMEA104679569	GCA_900312965	
Corynebacterium belfantii FRC0043	Dazas et al. 2018	DSM 105776; CIP 111412	*Corynebacterium belfantii*	2598 827	53.6	2557	Gp0364753	PRJEB22103	SAMEA104208677	GCA_900205605	
Corynebacterium phocae DSM 44612	Pascual et al. 1998	CCUG 38205; JCM 12105; CIP 105741; strain M408/89/1	*Corynebacterium phocae*	2791 853	58.8	2246	Gp0118686	PRJNA242582	SAMN02996498	GCA_001941565	
Corynebacterium silvaticum KL0182	Dangel et al. 2020	LMG 31313; DSM 109166; CIP 111672	*Corynebacterium silvaticum*	2548 487	54.4	2017		PRJNA517029	SAMN10039578	GCA_004382825	
Corynebacterium pseudopelargi CCM 8832	Busse et al. 2019	812CH; LMG 30627; CCUG 72167	*Corynebacterium pseudopelargi*	2348 160	57.9	2199	Gp0379416	PRJNA224116	SAMN08449372	GCF_003814005	

Strain	Authority	Other deposits	Synonyms	Base pairs	Percent G+C	No. proteins	Goldstamp	Bioproject accession	Biosample accession	Assembly accession	IMG OID
Corynebacterium rouxii FRC0190 T	Badell et al. 2020	DSM 110354; CIP 111752	*Corynebacterium rouxii*	2451 019	53.2	2365		PRJNA224116	SAMEA5992727	GCF_902702935	
Corynebacterium pelargi DSM 46737	Kämpfer et al. 2015	136/3; LMG 28174; CCM 8517; CIP 110778	*Corynebacterium pelargi*	2370 060	58.2	2169	Gp0442046	PRJNA224116	SAMN06041739	GCA_004114895	
Corynebacterium pseudotuberculosis ATCC 19410	(Buchanan 1911) Eberson 1918 emend. Nouioui et al. 2018	CCUG 2806; DSM 20689; ATCC 19410; NCTC 3450; JCM 9389; IFO 15363; NBRC 15363; CIP 102968	*Bacillus pseudotuberculosis*; *Corynebacterium pseudotuberculosis*	2337 763	52.2	2146	Gp0223239	PRJNA382169	SAMN06701041	GCA_002155265	
Corynebacterium pseudotuberculosis DSM 20689	(Buchanan 1911) Eberson 1918 emend. Nouioui et al. 2018	CCUG 2806; DSM 20689; ATCC 19410; NCTC 3450; JCM 9389; IFO 15363; NBRC 15363; CIP 102968	*Bacillus pseudotuberculosis*; *Corynebacterium pseudotuberculosis*	2338 546	52.2	2084	Gp0220522	PRJNA442833	SAMN08778220	GCA_003634885	2756170169
Corynebacterium ulcerans NCTC 7910	(ex Gilbert and Stewart 1927) Riegel et al. 1995	CCUG 2708; DSM 46325; ATCC 51799; JCM 10387; CIP 106504	*Corynebacterium ulcerans*	2453 761	53.3	2178	Gp0262745	PRJEB6403	SAMEA4504038	GCA_900187135	

Strain	Authority	Other deposits	Synonyms	Base pairs	Percent G+C	No. proteins	Goldstamp	Bioproject accession	Biosample accession	Assembly accession	IMG OID
Corynebacterium kutscheri DSM 20755	(Migula 1900) Bergey et al. 1925 emend. Nouioui et al. 2018	CCUG 27535; ATCC 15677; NCTC 11138; JCM 9385; IFO 15288; NBRC 15288; CIP 103423	*Bacterium kutscheri*; *Corynebacterium kutscheri*	2354 065	46.5	2047	Gp0110293	PRJNA276037	SAMN03365283	GCA_000980835	
Corynebacterium mustelae DSM 45274	Funke et al. 2010 emend. Nouioui et al. 2018	3105; CCUG 57279	*Corynebacterium mustelae*	3474 226	52.6	3110	Gp0114696	PRJNA282348	SAMN03568800	GCA_001020985	
Corynebacterium diphtheriae NCTC 11397	(Kruse 1886) Lehmann and Neumann 1896 emend. Nouioui et al. 2018	DSM 44123; ATCC 27010; CIP 100721	*Bacillus diphtheriae*; *Corynebacterium diphtheriae*; *Corynebacterium diphtheriae* subsp. *diphtheriae*	2463 666	53.5	2337	Gp0132011	PRJEB6403	SAMEA2517360	GCA_001457455	
KL0475				2472 849	53.3	2279					
KL0483				2577 295	53.4	2395					
KL0497				2477 500	53.3	2261					
KL0501				2446 525	53.3	2221					
KL0515				2449 403	53.3	2212					
KL0540				2474 507	53.4	2254					
KL0541				2640 162	53.4	2485					
KL0547				2549 371	53.3	2364					
KL0556				2421 288	53.3	2169					
KL0785				2470 695	53.4	2237					

Strain	Authority	Other deposits	Synonyms	Base pairs	Percent G+C	No. proteins	Goldstamp	Bioproject accession	Biosample accession	Assembly accession	IMG OID
KL0796				2603 053	53.4	2435					
KL0818				2474 549	53.4	2245					
KL0825				2499 718	53.3	2289					
KL0832				2452 482	53.3	2217					
KL0840				2449 355	53.3	2212					
KL0846				2443 613	53.3	2205					
KL0853				2557 514	53.4	2343					
KL0867				2514 118	53.3	2319					
KL0870				2575 028	53.4	2385					
KL0876				2469 953	53.3	2231					

The genome sequence data were uploaded to the Type (Strain) Genome Server (TYGS), a free bioinformatics platform available under https://tygs.dsmz.de, for a whole genome-based taxonomic analysis [1]. The results were provided by the TYGS on 2020-06-11. In brief, the TYGS analysis was subdivided into the following steps:

Determination of closely related type strains

Determination of closest type strain genomes was done in two complementary ways: First, all user genomes were compared against all type strain genomes available in the TYGS database via the MASH algorithm, a fast approximation of intergenomic relatedness [2], and, the ten type strains with the smallest MASH distances chosen per user genome. Second, an additional set of ten closely related type strains was determined via the 16S rDNA gene sequences. These were extracted from the user genomes using RNAmmer [3] and each sequence was subsequently BLASTed [4] against the 16S rDNA gene sequence of each of the currently 11820 type strains available in the TYGS database. This was used as a proxy to find the best 50 matching type strains (according to the bitscore) for each user genome and to subsequently calculate precise distances using the Genome BLAST Distance Phylogeny approach (GBDP) under the algorithm 'coverage' and distance formula d_5 [5]. These distances were finally used to determine the 10 closest type strain genomes for each of the user genomes.

Pairwise comparison of genome sequences

All pairwise comparisons among the set of genomes were conducted using GBDP and accurate intergenomic distances inferred under the algorithm 'trimming' and distance formula d_5 [5]. 100 distance replicates were calculated each. Digital DDH values and confidence intervals were calculated using the recommended settings of the GGDC 2.1 [5].

Phylogenetic inference

The resulting intergenomic distances were used to infer a balanced minimum evolution tree with branch support via FASTME 2.1.4 including SPR postprocessing [6]. Branch support was inferred from 100 pseudo-bootstrap replicates each. The trees were rooted at the midpoint [7] and visualized with PhyD3 [8].

Type-based species and subspecies clustering

The type-based species clustering using a 70% dDDH radius around each of the 14 type strains was done as previously described [1]. The resulting groups are shown in Table 1 and 4. Subspecies clustering was done using a 79% dDDH threshold as previously introduced [9].

Results

Type-based species and subspecies clustering

The resulting species and subspecies clusters are listed in Table 4, whereas the taxonomic identification of the query strains is found in Table 1. Briefly, the clustering yielded 12 species clusters and the provided query strains were assigned to 1 of these. Moreover, user strains were located in 1 of 12 subspecies clusters.

Figure caption genome tree

Figure 1. Tree inferred with FastME 2.1.6.1 [6] from GBDP distances calculated from genome sequences. The branch lengths are scaled in terms of GBDP distance formula d_5. The numbers above branches are GBDP pseudo-bootstrap support values > 60 % from 100 replications, with an average branch support of 26.8 %. The tree was rooted at the midpoint [7].

Figure caption SSU tree

Figure 2. Tree inferred with FastME 2.1.6.1 [6] from GBDP distances calculated from 16S rDNA gene sequences. The branch lengths are scaled in terms of GBDP distance formula d_5. The numbers above branches are GBDP pseudo-bootstrap support values > 60 % from 100 replications, with an average branch support of 43.4 %. The tree was rooted at the midpoint [7].

References

[1] Meier-Kolthoff JP, Göker M. TYGS is an automated high-throughput platform for state-of-the-art genome-based taxonomy. Nat. Commun. 2019;10: 2182. DOI: 10.1038/s41467-019-10210-3

[2] Ondov BD, Treangen TJ, Melsted P, et al. Mash: Fast genome and metagenome distance estimation using MinHash. Genome Biol 2016;17: 1–14. DOI: 10.1186/s13059-016-0997-x

[3] Lagesen K, Hallin P. RNAmmer: consistent and rapid annotation of ribosomal RNA genes. Nucleic Acids Res. Oxford Univ Press; 2007;35: 3100–3108. DOI: 10.1093/nar/gkm160

[4] Camacho C, Coulouris G, Avagyan V, Ma N, Papadopoulos J, Bealer K, et al. BLAST+: architecture and applications. BMC Bioinformatics. 2009;10: 421. DOI: 10.1186/1471-2105-10-421

[5] Meier-Kolthoff JP, Auch AF, Klenk H-P, Göker M. Genome sequence-based species delimitation with confidence intervals and improved distance functions. BMC Bioinformatics. 2013;14: 60. DOI: 10.1186/1471-2105-14-60

[6] Lefort V, Desper R, Gascuel O. FastME 2.0: A comprehensive, accurate, and fast distance-based phylogeny inference program. Mol Biol Evol. 2015;32: 2798–2800. DOI: 10.1093/molbev/msv150

[7] Farris JS. Estimating phylogenetic trees from distance matrices. Am Nat. 1972;106: 645–667.

[8] Kreft L, Botzki A, Coppens F, Vandepoele K, Van Bel M. PhyD3: A phylogenetic tree viewer with extended phyloXML support for functional genomics data visualization. Bioinformatics. 2017;33: 2946–2947. DOI: 10.1093/bioinformatics/btx324

[9] Meier-Kolthoff JP, Hahnke RL, Petersen J, Scheuner C, Michael V, Fiebig A, et al. Complete genome sequence of DSM 30083^{T}, the type strain (U5/41^{T}) of *Escherichia coli*, and a proposal for delineating subspecies in microbial taxonomy. Stand Genomic Sci. 2014;9: 2. DOI: 10.1186/1944-3277-9-2
